# RNA Interference-Based Genetic Engineering Maize Resistant to *Apolygus lucorum* Does Not Manifest Unpredictable Unintended Effects Relative to Conventional Breeding: Short Interfering RNA, Transcriptome, and Metabolome Analysis

**DOI:** 10.3389/fpls.2022.745708

**Published:** 2022-02-24

**Authors:** Chunmeng Huang, Zhi Wang, Pengyu Zhu, Chenguang Wang, Chaonan Wang, Wenjie Xu, Zhihong Li, Wei Fu, Shuifang Zhu

**Affiliations:** ^1^College of Plant Protection, China Agricultural University, Beijing, China; ^2^Chinese Academy of Inspection and Quarantine, Beijing, China

**Keywords:** RNAi-based genetic engineering maize, unintended effect, insect-resistant, biosafety assessment, transcriptome, metabolome

## Abstract

The use of omics techniques to analyze the differences between genetic engineering organisms and their parents can identify unintended effects and explore whether such unintended effects will have negative consequences. In order to evaluate whether genetic engineering will cause changes in crops beyond the changes introduced by conventional plant breeding, we compared the extent of transcriptome and metabolome modification in the leaves of three lines developed by RNA interference (RNAi)-based genetic engineering and three lines developed by conventional breeding. The results showed that both types of plant breeding methods can manifest changes at the short interfering RNA (siRNA), transcriptomic, and metabolic levels. Relative expression analysis of potential off-target gene revealed that there was no broad gene decline in the three RNAi-based genetic engineering lines. We found that the number of DEGs and DAMs between RNAi-based genetic engineering lines and the parental line was less than that between conventional breeding lines. These unique DEGs and DAMs between RNAi-based genetic engineering lines and the parental lines were not enriched in detrimental metabolic pathways. The results suggest that RNAi-based genetic engineering do not cause unintended effects beyond those found in conventional breeding in maize.

## Introduction

The application of genetic engineering (GE) technology to develop new crops with excellent biological characteristics was one of the strategies to ensure food security in the 21st century. To guarantee global food security, expert practitioners have applied biotechnology to crop breeding to obtain many GE crops with disease and insect resistance, abiotic (salt, drought, cold, and heat) resistance, and nutritional improvement, and some of these crops that have undergone rigorous biosafety assessment have been widely planted in some countries worldwide [Ricroch et al., 2011; Li et al., 2020; International Service for the Acquisition of Agri-biotech Applications [ISAAA], 2021]. RNA interference (RNAi)-based insect-resistant crops were transferred into exogenous fragments of vital pest genes to produce double-stranded RNA (dsRNA), which was then cut into approximately 21–24 nt short interfering RNAs (siRNAs) in the plant using Dicer or Dicer-like proteins. By means of base pairing, these siRNAs can target mRNA sequences in pests and subsequently degrade for pest control (Mamta and Rajam, 2017). RNAi can be used in the “in-species” mode of plant genomes to improve nutritional content by reducing antinutrients, allergens, and toxins while increasing the level of beneficial nutrients and inhibiting the growth of undesirable traits to improve productivity (Ramon et al., 2014). Similarly, RNAi can be used in plants to express dsRNA derived from genes outside the parental plant. Virus-resistant and insect-resistant crops obtained using RNAi have been approved for cultivation, including plums (*Prunus salicina*), soybeans (*Glycine max*), maize (*Zea mays*), Cassava (*Manihot esculenta*), and apples (*Malus pumila*).^1^ The public’s concerns mainly focus on potential risks to the environment and human health, contributing to delayed commercialization of GE products in many countries (Li et al., 2020). Therefore, before any new GE products have been allowed to enter the market, rigorous safety assessment research was crucial, the purpose of which was to identify and avoid risks.

The potential risks of GE crops have been separated into two broad categories: intended and unintended changes (Ladics et al., 2015). The assessment of intended changes uses measurement indicators such as molecular and biological characteristics and crop phenotypes, while there is no uniform standard for the measurement of unintended changes (Ladics et al., 2015). We can obtain specific biological traits or expected phenotypic traits by transferring specific exogenous genes into recipient crops using GE technology, however, we cannot guarantee that the exogenous gene will be integrated into the specific location of the recipient genome, which may lead to unintended integration, such as undesirable integration location, copy number, etc. This unintended integration often causes the final phenotypic change, but the trace compounds that caused the phenotypic change may not be detected downstream of the breeding process, which these trace amounts of compounds may affect nutrition and quality, even related to allergic and toxic effects (Zhao and Wolt, 2017). Based on this, two unintended risk assessment strategies have been suggested. The first strategy is to collect data on the phenotypic characteristics of GE crops including measuring the overall phenotype of crops and the composition of principal tissues, transcriptome, proteome, and metabolome (Ladics et al., 2015). Unintended changes are identified by comparing these data between GE crops and their counterparts (Bregitzer et al., 1998; Harrigan et al., 2010). The second strategy uses targeted sequencing and non-targeted omics data analysis of possible unintended omics data changes (Ladics et al., 2015). The latter approach considers risk assessment to be a hypothesis-driven test more comprehensively and scientifically reflecting differences between GE crops and their comparable counterparts (Ladics et al., 2015). Guided by relevant legislation and regulation (Evans et al., 2006), risk assessment experts determine the possible unintended effects and establish a series of scenarios that may be caused by specific events, excluding extreme and scientifically unreliable assumptions after the fact, determine the probability of these events and their frequency or magnitude, and collect data to test these hypotheses and characterize risks, including unintended effects (Ladics et al., 2015). The risk assessment based on tests and hypotheses can minimize unintended risks in GE crops (Ladics et al., 2015). Transcriptome, proteomics, and metabolomics have been used to evaluate the unintended effects of GE crop breeding at the mRNA, protein, and metabolite levels (Ricroch et al., 2011). Most such studies use one or two omics techniques to investigate the unintended effects, focusing on the comparison between GE lines and parental line, revealing some degree of variation. These studies cannot entirely and clearly establish whether the detected variation comes from insertion of exogenous gene or from the environment or genetic background or if they are attributed to normal variation in conventional breeding lines (Wang et al., 2019). At present, some have proposed that omics evaluation experiments on GE plants should not only establish parental control lines but also should use conventional breeding lines as controls for comparative analysis, which is a highly recognized approach (Klumper and Qaim, 2014).

Many things can induce unintended effects, which may emerge at any stage of the GE plant development process, including random mutations, somatic mutations, gene sequence insertions, positional effects, inductive effects, mutations in the tissue culture process, and pleiotropy (Weng et al., 2019). However, RNAi-based GE crops have remarkable molecular characteristics compared with conventional GE crops. If siRNAs were highly matched with non-target sequences to produce inhibitory effects, they may have unintended off-target effects although non-target species may include the GE plants themselves. The possible impact of siRNAs on plant genomes has not been clarified. The European Food Safety Agency’s GE management team believes that the relevant safety assessment content provided by research and development (R&D) applicants must be as detailed as possible and emphasize the importance of bioinformatics in the process of off-target effect analysis (Papadopoulou et al., 2020).

**FIGURE 1:**
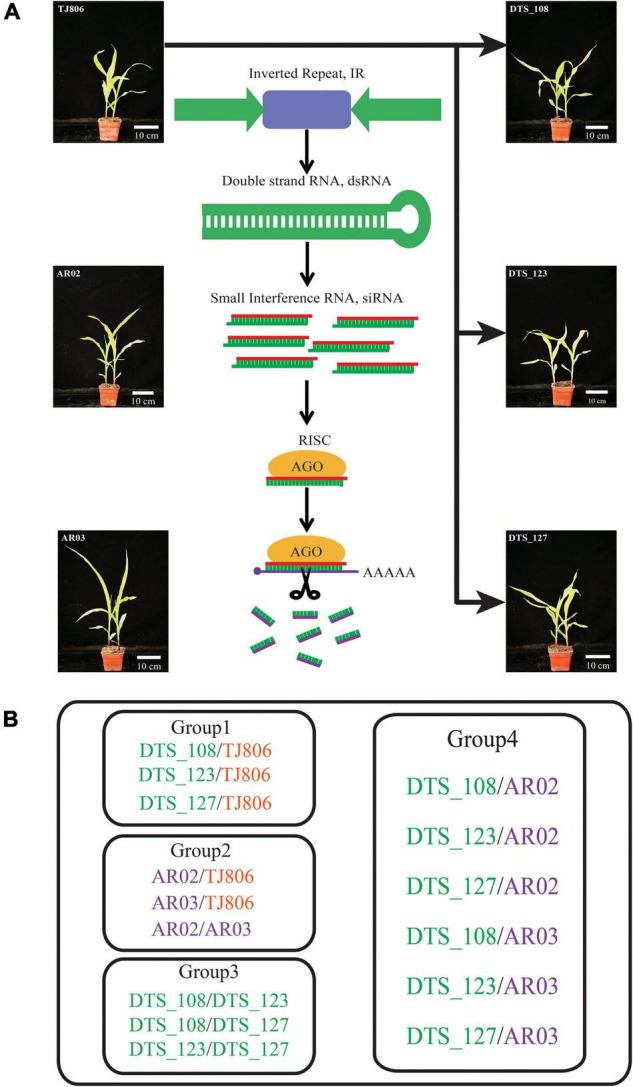
Genetic relations among the studied maize lines and grouping comparison design for the analyses. **(A)** Genetic relations among the studied maize lines. **(B)** Experimental design for pairwise comparisons of short interfering RNA (siRNA) expression, gene expression, and metabolite accumulation between different maize lines. Group 1, comparisons between RNA interference (RNAi)-based genetic engineering (GE) lines and parental line. Group 2, comparisons between conventional breeding lines and the non-GE parental line. Group 3, comparisons between RNAi-based GE lines with the same parents. Group 4, comparisons between RNAi-based GE lines and conventional breeding lines. DTS_108, DTS_123, and DTS_127 were RNAi-based GE lines transformed using the conventionally bred maize line TJ806. Maize lines with green, orange, and purple colors represent the RNAi-based GE lines, parental line of RNAi-based GE lines, and conventional bred maize lines, respectively.

Maize, one of the most important food, feed, and energy crops in the world, is damaged by pests, and diseases bring massive losses to farmers (Li et al., 2020). The data show that the four countries with the most GE crops have planted large areas of GE maize (International Service for the Acquisition of Agri-biotech Applications [ISAAA], 2021). In the context of increased resistance to individual Bacillus thuringiensis (Bt) target pests, GE plants expressing dsRNA represent a new generation of GE plants, but there has been little research on safety assessments of the unintended effects of RNAi-based GE crops (Casacuberta et al., 2015; Mamta and Rajam, 2017). In this study, RNAi-based GE lines (DTS_108, DTS_123, DTS_127) resistant to *Apolygus lucorum* (Meyer-Dür) (Hemiptera: Miridae) (reference patent No. US9944948B2), the parental line (TJ806), and conventional breeding lines (AR02 and AR03) were used to compare and evaluate unintended effects. We compared biological variations among the six maize lines at the siRNA, mRNA, and metabolite levels (Figure 1). The research constructed the datasets using omics-based systems biology methods, including siRNA sequencing, transcriptomics using RNA-seq, and metabolomics using high-performance liquid chromatography mass spectrometry (HPLC-MS). Based on these results, the potential unintended effects caused by two different plant breeding methods were analyzed comparably.

## Materials and Methods

### Materials

In this study, we used a total of six maize lines, including three RNAi-based GE lines (DTS_108, DTS_123, DTS_127), one parental line (TJ806), and two conventional breeding lines (AR02 and AR03). Three RNAi-based GE lines resistant to *A. lucorum* were different transformants containing the same exogenous inverted repeat sequences and transformed from the same parental line TJ806 (Figures 1A,B and Supplementary Table 1). Three RNAi-based GE lines have completed the intermediate test stage of biosafety evaluation and were about to enter the stage of environmental release. AR03 was a derivative line of AR02, among which AR02 was the hybrid parent of AR03. The genetic relationship between these two lines was similar, and both have been cultivated in China for several years. The parental line TJ806 has no genetic relationship with conventional breeding lines AR02 and AR03. We set up the comparison between the RNAi-based GE lines and parental line, including DTS_108/TJ806, DTS_123/TJ806, and DTS_127/TJ806, which belong to group 1. We also set up the comparisons between parental line and conventional breeding lines, including AR02/TJ806, AR03/TJ806, and AR02/AR03, which belong to group 2. The comparisons were also set up between GE lines, including DTS_108/DTS_123, DTS_108/DTS_127, and DTS_123/DTS_127, which belong to group 3. The comparisons between GE lines and conventional breeding lines, including DTS_108/AR02, DTS_123/AR02, DTS_127/AR02, DTS_108/AR03, DTS_123/AR03, and DTS_127/AR03, were set up, which belong to group 4. A total of 15 pariwise comparisons were set up as shown in Figure 1B. In order to make the analysis more clear, we divided the 15 pariwise comparisons into four groups, which represent the differences between RNAi-based GE lines and parental line (group 1), the differences between conventional breeding lines and parental line (group 2), the differences between GE lines (group 3), and the differences between GE lines and conventional breeding lines (group 4) (Figures 1A,B and Supplementary Table 1). All materials were contributed by Dabeinong Biotechnology Co., Ltd (Haidian, Beijing, China).

### Plant Growth Conditions and Tissue Sampling

The surface-sterilized rice seeds were germinated on half-strength MS medium. After a week, we transplanted the seedlings into individual plastic pots and placed all potted plants in a cement pool maintained in a glasshouse (28 ± 2°C RT, 65 ± 10% RH, 16 h light/8 h dark). After 5 weeks, from each plant, we sampled a leaf section (approximately 2 cm) from the middle part of the second leaf blade from the top. The samples from six plants were pooled as one biological replicate, and three replicates were collected for each maize line. The leaf samples were immediately frozen in liquid nitrogen and stored at −80°C.

### Small RNA Extraction, Library Preparation, and Small RNA Sequencing

To construct libraries for small RNA sequencing, we extracted and purified small RNA from maize leaves using RNAi-so Plus system and BioAnalyser 2100. The cDNA synthesis was carried out using small RNA with linkers as a template, and the small RNA library was constructed by cDNA after 15 PCR cycles. Library sequencing is based on the method of sequencing by synthesis using Illumina HiSeq 2000. We generated the original fastq file data using primers and vector sequences, subjected them to quality inspection and length screening of the sequenced fragment bases, and finally selected reliable small RNA sequencing fragments with lengths of 18–30 nt.

### Mock Library Construction, Sequence Alignment, and Verification of Gene Expression Level

The inverted repeat sequence with a length of 876 bp mainly derived from *A. lucorum* was used for library construction. We used Jellyfish (v.2.2.5) software to cut candidate sequences from different starting positions (first, second, etc.) of the dsRNA sequence, assembling a 21–24 bp kmer sequence library, and used Bowtie (v.1.1.0) software to compare it with maize transcripts to determine the start and end positions of each kmer. The mismatch parameter was set to within two bases.

First, we compared the siRNA in the mock library to the maize transcript (B73 RefGen_v4). The alignment setting allows two gaps to obtain specific maize transcripts (the potential off-target genes in maize). Second, we constructed the siRNA library by comparing actual small RNA sequencing to dsRNA sequence. To ensure that the analyzed siRNAs were all derived from the inverted repeat sequence inserted from an external source, we set the alignment threshold to 0 gaps. The results of the two comparisons can be combined to obtain the number of genes that siRNA has compared to the maize transcript. Base preference was profiled with WebLogo (Crooks et al., 2004). In addition, the distribution of siRNAs highly enriched on the dsRNA was determined with Grahprism7.0 software. These genes were used as potential off-target genes for further analysis, and their expression levels were measured using qRT-PCR calculated by 2^–ΔΔCt^ method. The experiment was designed in three parallels, and the significance between gene expression levels was tested using a student *t*-test (*p* < 0.05).

### Total RNA Extraction, Library Preparation, and RNA Sequencing for Transcriptome

Total RNA per samples was extracted using NEBNext^®^ UItra™ RNA Library Prep Kit for Illumina^®^ (NEB, United States). In total, 18 cDNA libraries, which were sequenced using Illumina HiSeq 4000 platform, were constructed. The clean reads were aligned to the reference genome AGPv4 using Bowtie2 software.^2^ The expression levels of all transcripts from the six leaves were quantified as the fragments per Kb per million reads (FPKM) (Trapnell et al., 2010) using the omicshare platform.^3^ The DEGs were identified using Benjamini and Hochberg’s approach with an adjusted *P*-value less than 0.05 and a fold change ≥2 or ≤0.5. The verification method of DEGs was same as described in Section “Mock Library Construction, Sequence Alignment, and Verification of Gene Expression Level.” The heatmap of relative expression level of the DEGs was plotted using the omicshare platform. Gene ontology (GO) and Kyoto encyclopedia of genes and genomes (KEGG) pathway enrichment analyses were performed using omicshare platform and MetaboAnalyst 4.0 (Chong et al., 2018) with a false discovery rate (FDR) adjusted *P*-value < 0.05 (hypergeometric test).

### Metabolite Profiling

#### Metabolites Extraction Process

After grinding six maize samples with liquid nitrogen, we added 400 μl of precooled methanol/acetonitrile/water solution (4:4:2, v/v) to the samples (Mix, stand at −20°C for 60 min, centrifuged at 14,000*g* at 4°C for 20 min). We vacuum dried the supernatant, added 100 μL of acetonitrile aqueous solution (acetonitrile: water = 1:1, v/v) to reconstitute during mass spectrometry, vortexed, centrifuged at 14,000*g* at 4°C for 15 min, and took 2 μL of supernatant for sample analysis.

#### High-Performance Liquid Chromatography and Electrospray Ionization-Q trap-MS/MS Running Conditions

The samples were separated using an Agilent 1290 Infinity LC Ultra HPLC system (UHPLC) (HILIC column temperature 25°C, flow rate 0.3 mL/min, injection volume 2 μL) following the manufacturer’s instructions. The sample was placed for the entire analysis process in the autosampler at 4°C. We adopted a random order for continuous analysis of the samples.

We used electrospray ionization (ESI) positive ion and negative ion modes for detection. We separated the samples using UHPLC and analyzed them with a Triple TOF 6600 mass spectrometer (AB SCIEX) following the manufacturer’s instructions from Hoogen biotech Co., Ltd (Minhang, Shanghai, China).

#### Acquiring Metabolic Data

The original data to mzXML format using Proteo Wizard then used the XCMS program for peak alignment, retention time correction, and peak area extraction. The metabolite structure identification uses accurate mass matching (<25 ppm) and secondary spectrum matching methods and searches the laboratory’s self-built database. For the data extracted by XCMS, ion peaks with missing values >50% were deleted in the group. The application software SIMCA-P 14 (Umetrics, Umea, Sweden) was used for pattern recognition and the data were preprocessed by Pareto-scaling for multidimensional statistical analysis.

#### Data Analysis

According to the variable weight value, variable importance for the projection (VIP), obtained by the orthogonal partial least squares discriminant analysis (OPLS-DA) model, the influence intensity and explanatory power of the expression pattern for each metabolite can be measured. Those with fold change (FC) > 2.0 and *P*-value < 0.05 were used as differential cumulative metabolites. Using qualitatively significant differences in metabolite expression levels for each group of samples, the clustering (hierarchical clustering) helps us to accurately screen marker metabolites and use the KEGG public database to conduct pathway analysis of differential metabolites.

## Results

### Evaluating Omic Data Derived From Leaves From Six Maize Lines

We conducted siRNA-seq, RNA-seq, and metabolite analysis to investigate unintended effects in RNAi-based maize. For siRNAs, we constructed a total of 18 small RNA libraries resulting in approximately 15.84–23.80 million raw reads per library and 10.34–11.98 million clean reads per library being cleated. The rate of clean reads ranged from 50.54 to 83.51%. The percentage of bases with a Phred value greater than 30 compared to total bases was between 95.73 and 97.50% (Supplementary Table 2). For transcriptome analysis, the 18 RNA-seq libraries were constructed, resulting in approximately 51–56 million clean reads per library being cleated. Using the *Zey_mays* AGPv4 as a reference genome, 92.05–96.75% of the clean reads were mapped. The percentage of bases with a Phred value greater than 30 compared to total bases was between 94.06 and 94.55% (Supplementary Table 3). We profiled metabolic changes in the six maize lines. We detected a total of 1,954 metabolites (Supplementary Table 4). The majority of these metabolites belonged to different ontologies, i.e., diterpenoids, phenolic glycosides, and alpha amino acids and derivatives (Supplementary Table 4). These results suggest that the datasets generated from these six maize lines were sufficient for further analyses.

### Features of Short Interfering RNAs of Six Maize Lines

**FIGURE 2:**
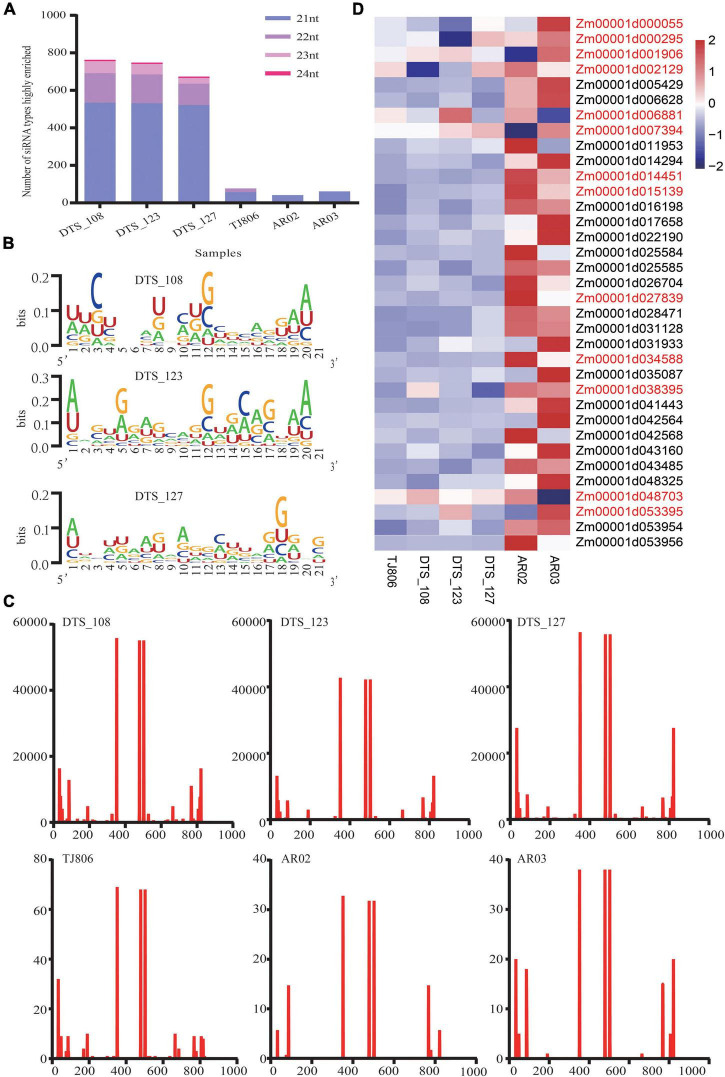
The features of siRNAs highly enriched in maize and expression of potential off-target genes. **(A)** The number of siRNA types with lengths of 21–24 nt highly enriched in six maize lines. **(B)** The distribution of the first base of siRNAs in double-stranded RNA (dsRNA) sequences in six maize lines. The abscissa indicates the base position of the dsRNA sequence. The ordinate indicates that the number of siRNA reads was highly enriched in six maize lines. **(C)** The base preference of siRNAs with a length of 21 nt highly enriched in three RNAi-based GE lines. The abscissa indicates the base position of siRNAs with a length of 21 nt. The ordinate indicates the proportion of bases (A/U/G/C) in each base position. Larger bases represent a higher frequency of bases. **(D)** Heatmap of the expression levels of 35 potential off-target genes in six maize lines. Gene names colored red indicates that a gene was expressed differentially in maize lines (*t*-test, *p* < 0.05).

We analyzed the lengths of highly enriched siRNAs, base preferences, and their distributions on the dsRNA sequence (Figure 2 and Supplementary Tables 2–4). The lengths of highly enriched siRNAs were concentrated at 21 nt in all maize lines (Supplementary Table 5). The siRNA size distribution shown in Figure 2A was comparable with previous observations in Arabidopsis that the 21 nt long siRNA is the predominant antiviral silencing component. Robust guanine and cytosine (GC) bias (52.75%) was observed for all siRNAs highly enriched in the maize transcriptome. The adenine or uracil (A/U) content of five bases at the 5′ end of siRNAs was higher than the A/U content of other positions (Figure 2B and Supplementary Table 6), related to the binding stability of the siRNA targeting mRNA and one of the conditions for effective siRNA silencing. We analyzed the distribution of siRNAs and noted the positions of their first bases in the dsRNA sequence, with specific distribution characteristics present in six maize lines, although the distribution trend of siRNAs was similar (Figure 2C and Supplementary Table 7). Notably, the number of reads of siRNAs in RNAi-based GE lines was approximately 20,000 times greater than in non-GE maize lines.

### Verification of Potential Off-Target Gene Expression Levels Using RT-qPCR

We obtained 35 transcripts identified as potential off-target genes of maize. One gene (Zm00001d014294) was mapped to the transcriptome of maize when a 1 bp mismatch was set. Thirty-five genes were mapped to the transcriptome of maize when a 2 bp mismatch was set (Supplementary Table 6). Using *zssIIb* as an internal reference gene, RT-qPCR was used to analyze the relative expression of potential off-target genes, as shown in the heatmap (Figure 2D). The expression levels of the Zm00001d001906 and Zm00001d007394 genes in AR02 were significantly lower than those in DTS_127. The genes whose expression levels in AR02 were higher than those in AR03, DTS_123, TJ806, and DTS_127 were Zm00001d014451, Zm00001d015139, Zm00001d027839, and Zm00001d034588, respectively. The expression level of Zm00001d048703 in AR03 was significantly lower than in DTS_123. The expression patterns of these genes in specific strains were completely consistent with the expression patterns of DEGs in the transcriptome. In addition, compared with the parental line, the expression of genes Zm00001d000055 and Zm00001d000295 decreased in DTS_123. Although the expression levels of these potential off-target genes were lower than those in TJ806, this did not appear in all RNAi-based GE lines (Figure 2D).

### Analyzing Gene Expression Through RNA-Sequencing

**FIGURE 3:**
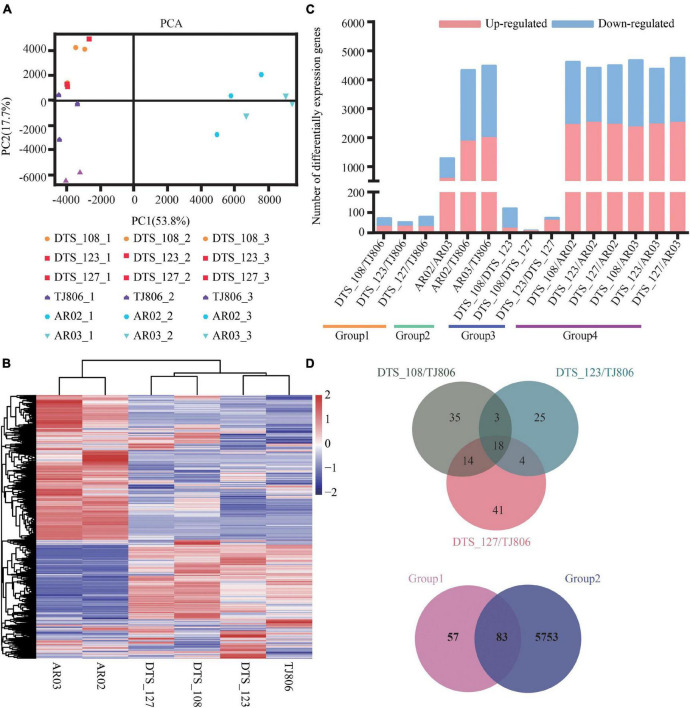
Overall description of transcriptome data. **(A)** Principal component analysis (PCA) of gene expression levels in the leaves of six maize lines. Score plot of the first two principal components with the explained variance. **(B)** Hierarchical clustering of six maize lines using the total detected gene expression data. In the heatmap, each maize line is visualized in a single column and each gene is represented by a single row. Gene expression levels are shown in different colors, where red indicates high abundance and low relative expression is shown in blue (color key scale right of the heatmap). **(C)** Pairwise comparisons of DEGs between different maize lines. **(D)** Venn diagrams depicting the unique and shared DEGs between lines obtained by RNAi-based genetic modification and conventional breeding methods.

The RNA-seq dataset was normalized to FPKM values to quantify the levels of gene expression, including 28,852 genes (Supplementary Table 8). The principal component analysis (PCA) was performed on all 18 transcriptomic datasets. As shown in Figure 3A, the first two principal components (PCs) explain 53.8% (PC1) and 17.7% (PC2) of total variance. PC1 revealed a clear separation between conventional breeding lines and RNAi-based GE lines compared with the parental line. However, the first two PCs failed to separate the GE lines from the parental line. Consistently, RNAi-based GE lines and conventional breeding lines were hierarchically clustered in the respective classes. Three RNAi-based GE lines had a closer genetic relationship with parental line (Figure 3B).

Subsequently, differentially expressed genes (DEGs) of the six maize lines based on different grouping comparisons as described in Figure 1 were screened, showing distinct differences in gene expression among the lines. A total of 8–4765 DEGs were detected among the 15 pairwise comparisons (Figure 3C and Supplementary Table 9). The number of DEGs between RNAi-based GE lines and parental line was less than between conventionally breeding lines. The number of DEGs between RNAi-based GE lines and parental line was within the normal variation of gene expression changes in conventionally breeding lines. The number of DEGs between RNAi-based GE lines and conventionally breeding lines was similar to that between parental line and conventionally breeding lines except for AR02/AR03, which is far more than the number of DEGs between RNAi-based GE lines and parental line, suggesting that different genetic backgrounds may bring more changes in gene expression (Figure 3C).

We calculated the distribution of DEGs for each comparison and present them in Venn diagrams (Figure 3D). This distribution was genotype-specific. As shown in Figure 3D, we analyzed the distribution of DEGs between three GE lines and parental line. The results showed that there were 35, 25, and 41 unique DEGs in DTS_108, DTS_123, and DTS_127, respectively. At the same time, three GE lines shared 18 DEGs compared with parental line (Figure 3D). Although a large number of DEGs were detected in pairwise comparisons, they still shared 83 DEGs between group 1 and group 2. There were 57 and 5753 unique DEGs in group 1 and group 2 representing the different breeding lines, respectively (Figure 3D). The number of unique DEGs in each RNAi-based GE line compared with group 2 was similar and was significantly less than that in group 2 (Supplementary Figure 1). These results suggest that both conventional and GE breeding methods can change the expression of non-target genes. We verified the expression levels of 20 DEGs from the shared and unique collection described above using RT-qPCR. As shown in the Supplementary Figure 1, which were RNA-seq and qPCR data, respectively. The results of heatmap showed that the expression trend of DEGs was consistent with the transcriptome sequencing data, indicating that the transcriptome sequencing results were accurate and credible.

### Functional Enrichment Analysis of Differentially Expressed Genes

Gene ontology pathway enrichment analyses of the DEGs in a total of 15 pairwise group comparisons were conducted (Supplementary Table 10). No significantly enriched biological process GO terms were found in comparisons between RNAi-based GE lines and parental line (Supplementary Table 10). Different biological process terms were enriched in specific comparisons, with hydrolase activity acting on glycosyl bonds as the most popular pathway terms in the group 2, group 3, and group 4. We performed GO pathway enrichment analyses of the unique and shared DEGs of comparisons (not attached). Interestingly, there were zero and 43 significantly enriched GO terms of unique DEGs in group 1 and group 2, respectively. Only cytoskeletal terms were significantly enriched GO terms of unique DEGs in the comparisons of DTS-123/TJ806 when compared with group 2 and none of those in DTS-108/TJ806 and DTS-127/TJ806. The performances of the three RNAi-based maize lines were similar, with the number of enrichment pathways of unique DEGs far less than those in group 2 (not attached).

Similarly, the KEGG enrichment analyses indicated that there were no significantly enriched pathways for the comparisons between RNAi-based GE lines and parental line except for the eukaryotic ribosome biogenesis pathway in the comparison of DTS_123/TJ806. In addition, there were some pathways, such as ABC transporters, Diterpenoid biosynthesis, Flavonoid biosynthesis, Monoterpenoid biosynthesis, Phenylpropanoid biosynthesis, and Plant hormone signal transduction, which were significantly enriched in the comparisons between conventionally breeding lines with a high enrichment score. There were Photosynthesis, Flavonoid biosynthesis, Cyanoamino acid metabolism, Starch and sucrose metabolism, Ribosome biogenesis in eukaryotes, and Phenylpropanoid biosynthesis that were significantly enriched in the comparisons between the RNAi-based GE lines and conventionally breeding lines (Table 1).

**TABLE 1:** Kyoto encyclopedia of genes and genomes (KEGG) pathway enrichment analysis of significantly DEGs.

Group	Comparisons	Id	Term	ListHits	ListTotal	PopHits	PopTotal	pval	padj	Enrichment_score
Group 1	DTS_108/TJ806	-								
	DTS_123/TJ806	path:zma03008	Ribosome biogenesis in eukaryotes	2	44	49	6694	0.0040	0.0287	6.2096
	DTS_127/TJ806	-								
Group 2	AR02/TJ806	path:zma00500	Starch and sucrose metabolism	26	771	112	6694	0.0001	0.0146	2.0155
		path:zma02010	ABC transporters	2	44	15	6694	0.0001	0.0033	20.2848
		path:zma00902	Monoterpenoid biosynthesis	4	771	7	6694	0.0003	0.0201	4.9613
	AR03/TJ806	path:zma00500	Starch and sucrose metabolism	26	771	112	6694	0.0001	0.0146	2.0155
		path:zma00941	Flavonoid biosynthesis	2	63	27	6694	0.0020	0.0098	7.8707
		path:zma00940	Phenylpropanoid biosynthesis	5	63	145	6694	0.0023	0.0098	3.6639
		path:zma00480	Glutathione metabolism	21	809	74	6694	0.0000	0.0042	2.3481
	AR02/AR03	path:zma00904	Diterpenoid biosynthesis	2	63	16	6694	0.0004	0.0058	13.2817
		path:zma04075	Plant hormone signal transduction	6	63	202	6694	0.0027	0.0098	3.1561
Group 3	DTS_108/DTS_123	-								
	DTS_108/DTS_127	-								
	DTS_123/DTS_127	-								
Group 4	DTS_108/AR02	path:zma00940	Phenylpropanoid biosynthesis	8	100	145	6694	0.0003	0.0050	3.6932
	DTS_123/AR02	path:zma00196	Photosynthesis - antenna proteins	3	100	24	6694	0.0004	0.0050	8.3675
	DTS_127/AR02	path:zma00460	Cyanoamino acid metabolism	3	100	34	6694	0.0015	0.0100	5.9065
		path:zma00941	Flavonoid biosynthesis	2	63	27	6694	0.0020	0.0098	7.8707
	DTS_108/AR03	path:zma00500	Starch and sucrose metabolism	5	63	112	6694	0.0006	0.0058	4.7435
	DTS_123/AR03	-								
	DTS_127/AR03	path:zma03008	Ribosome biogenesis in ryeukaotes	2	63	49	6694	0.0108	0.0251	4.3369
		path:zma00500	Starch and sucrose metabolism	7	100	112	6694	0.0002	0.0050	4.1838

### Metabolomic Differences in Leaves Among Maize Lines

**FIGURE 4:**
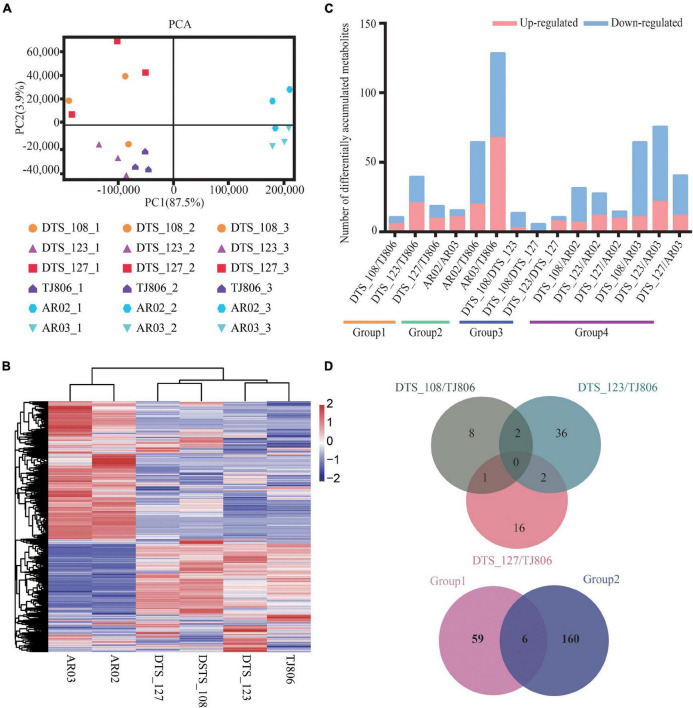
Overall description of metabolome data. **(A)** Principal component analyses (PCA) of metabolite accumulation levels in leaves of six maize lines. Score plot of the first two principal components with the explained variance. **(B)** Hierarchical clustering of six maize lines using the total detected metabolite accumulation data. In the heatmap, each maize line is visualized in a single column and each metabolite is represented by a single row. Metabolite accumulation levels are shown in different colors, where red indicates high abundance and low relative expression is shown in blue (color key scale right of the heatmap). **(C)** Pairwise comparisons of DAMs between different maize lines. **(D)** Venn diagrams depicting the unique and shared DAMs between lines obtained by RNAi-based genetic modification and conventional breeding methods.

A PCA plot for metabolite accumulation was constructed and shown in Figure 4A, where the abscissa and the ordinate represent the scores of PC1 and PC2, respectively. The first two PCs explain 87.5 and 3.9% of the total variance, respectively. PC1 showed a clear separation between maize lines with different genetic backgrounds. For specific maize lines, the first two PCs could not separate the GE lines from the parental line. Consistently, clustering analysis of the metabolites from the six maize lines showed that the conventional breeding lines and RNAi-based GE lines were clustered into distinct groups (Figure 4B).

The differentially accumulated metabolites (DAMs) in leaves among different maize lines were identified. A total of 6–129 DAMs were identified, ranging from 0.31 to 6.60% of the total detected metabolites in each of the 15 comparisons (Figure 4C and Supplementary Table 11). In addition, the number of DAMs between RNAi-based GE lines and parental line were, in all cases, less than those between RNAi-based GE lines and conventional breeding lines (Figure 4C and Supplementary Table 11). We constructed a venn diagram for qualitative analysis of the metabolites. The number of unique DAMs in DTS_108, DTS_123, and DTS_127 were 8, 36, 16, respectively, when compared with parental line (Figure 4D). It was noticed that there was no shared DAMs in three RNAi-based GE lines compared with parental line. Group 1 and group 2 shared six metabolites, with 59 and 160 unique metabolites, respectively (Figure 4D). Similar to the transcriptome results, the numbers of unique DAMs in the three RNAi-based maize lines were similar and far less than those in group 2 (Figure 4D).

The KEGG pathway enrichment analysis showed that no pathways were significantly enriched in the DAMs of group 1 and group 3. In contrast, some pathways were significantly enriched in the DAMs of group 2, including glycerolipid metabolism, selenocompound metabolism, galactose metabolism, alanine, aspartate and glutamate metabolism, aminoacyl-tRNA biosynthesis, and vitamin B6 metabolism. We found vitamin B6 metabolism to be enriched in DAMs between conventionally bred lines and the parental line of group 4 (Table 2). We profiled KEGG pathway enrichment analysis of significantly enriched shared or specific DAMs of comparisons of group 1 and group 2. Five KEGG pathways were significantly enriched in the unique DAMs of group 2 when compared with group 1, including vitamin B6 metabolism, ubiquinone and other terpenoid-quinone biosynthesis, nicotinate and nicotinamide metabolism, pyrimidine metabolism, and tyrosine metabolism. There was no significant enrichment of metabolites specific to group 1 or any metabolites specific to RNAi-based GE maize.

**TABLE 2:** Kyoto encyclopedia of genes and genomes (KEGG) pathway enrichment analysis of significantly DAMs.

Group	Pairwise comparison	KEGG pathway	Total	Expected	Hits	Raw *p*
Group 1	DTS_108/TJ806	-				
	DTS_123/TJ806	-				
	DTS_127/TJ806	-				
Group 2	AR02/AR03	Glycerolipid metabolism	16	0.021	1	0.0209
		Selenocompound metabolism	20	0.0263	1	0.0261
		Galactose metabolism	27	0.0355	1	0.0352
	AR02/TJ806	Alanine, aspartate, and glutamate metabolism	28	0.0368	1	0.0365
		Ubiquinone and other terpenoid-quinone biosynthesis	9	0.0296	1	0.0293
	AR03/TJ806	Vitamin B6 metabolism	9	0.0946	2	0.00357
Group 3	DTS-108/DTS-123	-				
	DTS-108/DTS-127	-				
	DTS-123/DTS-127	-				
Group 4	DTS_108/AR02	Vitamin B6 metabolism	9	0.0237	1	0.0235
	DTS-123/AR02	Ubiquinone and other terpenoid-quinone biosynthesis	9	0.0296	1	0.0293
	DTS-127/AR02	-				
	DTS_108/AR03	Vitamin B6 metabolism	9	0.0591	2	0.00137
	DTS_123/AR03	-				
	DTS_127/AR03	Vitamin B6 metabolism	9	0.065	2	0.00166
		Thiamine metabolism	7	0.0506	1	0.0496

## Discussion

In the 2000s, new methodologies were developed to allow, in theory, a holistic search for alterations in GE crops at different biological levels (transcripts, proteins, metabolites) (Ricroch et al., 2011). However, research on the assessment of unintended effects in RNAi-based GE crops, including off-target effect analysis, was little enough that their risk assessment has not been well-understood (Auer and Frederick, 2009). We attempted to determine the amount of variation among RNAi-based GE lines resistant to *A. lucorum* by establishing parental controls and conventional breeding lines using siRNA, mRNA, and metabolite data analysis.

Consistent with research on matching siRNA and target sequences, 21 nt siRNAs accounted for a large proportion of all siRNAs to be analyzed, indicating that among the 21–24 nt siRNAs, 21 nt siRNAs played a major role when siRNAs and non-target sequences were matched (Papadopoulou et al., 2020; Figure 2A). Interestingly, some potential off-target genes in RNAi-based GE lines with expression levels lower than those in the parental line were not common in all the three RNAi-based GE lines (Figure 2D). These results suggest that no off-target phenomenon was found by molecular experiments at the gene expression level. In fact, the gene expression levels are affected by many factors such as environment, weather, and varieties, possibly resulting in differences in gene expression and phenotypic changes (Zhang et al., 2017). The high abundance of siRNA in the maize genome did not possess an obvious inhibitory effect. This result is supported by previous studies showing that the off-target suppression effect does not solely depend on the abundance of siRNAs (Praveen et al., 2010; Papadopoulou et al., 2020). Whether off-target occurs other important factors must be considered such as the concentration of siRNAs in plant cells, the amount of siRNA loaded with AGO protein, and the binding energy between siRNA and its target mRNA. In the end, off-target effects should be verified by biological experiments (Ma et al., 2006; Papadopoulou et al., 2020). Therefore, the relationship among gene expression differences, phenotypic changes, and off-target effects should be explained carefully (Casacuberta et al., 2015; Ladics et al., 2015; Arpaia et al., 2020). Obviously, using bioinformatics to predict off-target genes is only a basic auxiliary approach, while the application of bioinformatics can provide a reference for off-target effects analysis (Ahmed et al., 2020; Papadopoulou et al., 2020).

Both PCA and hierarchical cluster analyses of the datasets showed a distinct separation between samples with different genetic backgrounds at both the transcriptome and metabolome levels. Specifically, there was a distinct separation between conventional breeding maize lines and RNAi-based GE lines (including parental line), but there was no separation trend between RNAi-based maize lines and the parental line (Figures 3A,B, 4A,B). This result was consistent with previous studies showing that different background varieties are clearly distinguishable but no distinction is seen between GE lines and parental line. Natural variation in plants is very common at the transcriptional and metabolic levels (Batista et al., 2008). These current results and previous studies suggest that intrinsic differences in genetic background bring much greater variation to the plant transcriptome and metabolome than the introduction of foreign genes by genetic manipulation or conventional breeding methods (Ladics et al., 2015; Wang et al., 2018).

As expected, pairwise comparisons reveal differences between the GE lines and parental line with respect to gene expression and metabolite accumulation, as reported previously for GE maize and soybeans (Liu et al., 2020). However, the number of DEGs and DAMs observed when comparing GE maize lines and the parental line were significantly less than those present when comparing conventional breeding lines and the parental line (Figures 3C, 4C; Wang et al., 2018). However, the number of DEGs in the transcriptome was not always consistent with the number of DAMs in the metabolome (Wang et al., 2019; Liu et al., 2020). Specifically, the number of DEGs between the parental line and the conventional breeding lines was close to the number of DEGs between the RNAi-based GE lines and the conventional bred lines but the performance of DAMs was different, indicating that genetic changes commonly occur during the plant breeding process whether done by GE or by conventional breeding, and the extent of those changes seems not always relevant to the extent of metabolomic changes in maize (Wang et al., 2019). The detection of DEGs and DAMs in RNAi-based GE plants was carried out under specific developmental or environmental conditions, which could yield unintended effects upon analysis. Assessment of unintended effects during specific developmental periods and conditions often ignores other factors, especially because these differences can affect gene transcription in GE plants (Herman and Price, 2013; Olmos et al., 2019). Therefore, it has been recommended that the unintended effects evaluation of GE plants should include a combination of omics to provide a parameter platform easier to understand and analyze (Ricroch et al., 2011; Liu et al., 2020).

We found that the number of shared DAMs between three GE lines and parental line was zero when we contacted the DEGs and DAMs between three GE lines and parental line indicating that we could not analyze whether there were common changes between the GE lines and parental line from the transcriptome and metabolome level. We therefore have tried to perform an association analysis on the unique DEGs and DAMs in group 1 and group 2, such as looking for DEGs that participated in expression regulation and can produce specific DAMs that belong to group 1 or group 2. Unfortunately, our analysis did not yield meaningful data. We thus analyzed the possible reasons for this result as follows. First, the number of DEGs and DAMs obtained by analyzing were too relatively small to support association strategy. Second, we did find some DEGs and DAMs between transgenic plants and parental line at the transcriptome level and metabolome level, respectively. However, these differences cannot be correlated from the transcriptome to the metabolome, which showed that none of the pathway from gene expression to metabolic regulation significantly altered; this result was consistent with the published research results (Ricroch et al., 2011). In addition, we found that these unique DEGs and DAMs have no same KEGG enrichment pathway. We speculate that the possible reason is that it is difficult to identify a pathway that significantly changes from gene expression to metabolic regulation, because it requires a lot of work to verify the key genes and their functions involved in the relevant biological pathways (Ricroch et al., 2011).

We did find some unique DEGs and DAMs in RNAi-based GE lines, although the number was significantly less than those in conventional breeding lines compared with the parental line (Figures 3D, 4D and Supplementary Tables 9, 11). This result implies that the process of GE may bring different stresses to the host genome relative to conventional breeding, indicating that the two plant breeding processes may result in variations in genes and metabolites at different levels (Hoekenga, 2008; Liu et al., 2020). Notably, there were more DEGs and DAMs in plants produced by conventional breeding than by genetic modification, possibly implying that the conventional breeding requires multiple crosses between two or more breeding lines, thus causing more variation at both genotypic and phenotypic levels. We used the substantial equivalence standard to assess the safety of food or feed produced by GE (Schiemann et al., 2019). We found some DAMs in the comparisons between RNAi-based GE plants and their parental counterparts, and these DAMs are also found in conventional breeding lines. Since these conventional breeding lines are considered to have a long history of use and safety, a control consisting of conventional breeding lines should be implemented when evaluating unintended effects (Chen et al., 2014). The European Food Safety Agency’s genetic modification management team has pointed out that the safety evaluation of GE plants includes GE near-isogenic control and reference commercial variety control (Eckerstorfer et al., 2019; Papadopoulou et al., 2020).

Based on the experimental data and results of siRNA, transcriptome, and metabolome, it can be concluded that RNAi-based GE maize is essentially equivalent to conventional breeding. The differences brought by GE breeding were not as obvious as those caused by conventional breeding, although conventional breeding also has some difference that cannot be explained clearly so far (Ricroch et al., 2011). It was important to keep in mind that the standard proposed by the OECD/Food and Agriculture Organization of the United Nations/WHO was substantial equivalence rather than total equivalence and that there was no specific statistical or biological basis to define “substantial” (Hoekenga, 2008). In other words, no “limits of concern” have been defined regarding differences. In order to make the conclusions more reliable, we considered the following points that may affect the data and even affect the risk assessment results of GE crops. One of the factors we need to pay attention to was the selection of experimental materials. We used leaves for testing mainly because RNAi-based GE maize was resistant to *A. lucorum*, which mainly damages leaves, that is, siRNA from *A. lucorum* plays a vital role in leaves. Meanwhile, the existing literatures have studied the unintended effects of GE crops using leaves (Wang et al., 2018; Liu et al., 2020). Furthermore, the gene expression and metabolism of plant leaves are active, which is very conducive to the collection of transcriptome and metabolome data. However, as grains of maize are the edible part, we should consider that the analysis of them may obtain more meaningful data. The leaves and grains or other reasonable research sample tissues should be considered in the future research. The second factor we considered was the number of samples. The prior probability refers to the probability obtained based on inference and observations when using omics technology to evaluate the unintended effects of GE crops. Increasing the number of test samples was a prerequisite for ensuring a higher prior probability (Ricroch et al., 2011). To obtain more accurate results, we set up three biological replicates of each of six maize lines to compare differences in gene expression and metabolite accumulation levels and each biological replicate is a mixture of 10 individual plant samples. We have chosen different omics methods to evaluate the unexpected effects of genetically modified crops such as siRNA, transcriptome, and metabolome since sample selection, sample numbers, and sampling location may affect the results of omics data. We found highly enriched siRNAs in the RNAi-based GE lines; however, we did not find that there were generally reduced potential off-target genes in all three RNAi-based GE lines during qPCR test. We note that not only eukaryotic ribosome biogenesis but also starch and sucrose metabolism, phenylpropanoid biosynthesis, and flavonoid biosynthesis were significantly enriched in the comparison of conventionally breeding maize lines and RNAi-based GE lines, indicating that the GE process resulted in DEGs and DAMs at the transcriptome and metabolome levels (Liu et al., 2020), but these pathways enriched in DEGs and DAMs were within the range of comparisons between conventional breeding lines and parental line (Wang et al., 2019; Liu et al., 2020). These results may thus suggest that the GE processes do not have unique effects on plant pathways compared with conventional breeding lines (Tables 1, 2 and Supplementary Table 12; Wang et al., 2019; Liu et al., 2020). If we can find some DEGs associated with DAMs in the transcriptome, the results will be easier to interpret, but this was not easy to achieve, although this was an ideal result. Thus, we propose a combination of multiple omics analysis, which can avoid the differences in the analysis of single omics data and explore as much as possible a metabolic pathway that regulated from gene expression to metabolites.

## Conclusion

In conclusion, we successfully employed siRNA-seq, RNA-seq, and HPLS-MS technology to investigate the changes in siRNA and gene expression and metabolite accumulation in six maize lines developed by RNAi-based GE or conventional breeding. We did find that the inverted repeat gene sequence from *A. lucorum* produced highly enriched siRNAs in GE maize lines. However, qRT-PCR and transcriptome data analysis showed that the decline in gene expression levels of these potential off-target genes was not universal in the three transgenic lines, meaning that the siRNA targeted for *A. lucorum* did not occur detectable gene suppression in maize, indicating that bioinformatics analysis can be used to determine which genes in non-target organisms have a certain degree of sequence homology with target genes. The current results showed that both GE and conventional breeding method can result in potential changes at transcriptome and metabolome levels and the GE does not cause unintended effects that go beyond conventional breeding. There was no pathway that significantly altered from gene expression to metabolic regulation involved in the study, suggesting that a comprehensive and comparative multi-omics sharing platform should be established to improve the effective utilization of data when assessing the unintended effect of GE crops.

## Data Availability Statement

The datasets presented in this study can be found in online repositories. The names of the repository/repositories and accession number(s) can be found below: https://www.ncbi.nlm.nih.gov/, PRJNA725413.

## Author Contributions

CH, ZW, ZL, WF, and SZ conceived the idea. CH, ZW, PZ, and CGW designed the study. CH, CNW, and WX performed the experiments. ZW and WX analyzed the data. CH and ZW wrote the manuscript. All authors have read and approved the manuscript for publication.

## Conflict of Interest

The authors declare that the research was conducted in the absence of any commercial or financial relationships that could be construed as a potential conflict of interest.

## Publisher’s Note

All claims expressed in this article are solely those of the authors and do not necessarily represent those of their affiliated organizations, or those of the publisher, the editors and the reviewers. Any product that may be evaluated in this article, or claim that may be made by its manufacturer, is not guaranteed or endorsed by the publisher.

## Footnotes

1
https://www.isaaa.org/gmapprovaldatabase/default.asp

2
http://tophat.cbcb.umd.edu/

3
https://www.omicshare.com/

## Supplementary Material

Supplementary Figure 1 Heatmap of verification results of DEGs. **(a)** Heatmap of verification results of DEGs for RNA-seq data. **(b)** Heatmap of verification results of DEGs for qPCR data.

Supplementary Figure 2 Venn diagram analysis between three GE lines and group 2 at the transcriptome and metabolome level. **(a–c)** Venn diagram analysis of DEGs between three GE lines and group 2 at the transcriptome level. **(d–f)** Venn diagram analysis of DAMs between three GE lines and group 2 at the metabolome level.

## Abbreviations

RNAi: RNA interference
dsRNA: double-stranded RNA
siRNAs: short interfering RNAs
HPLC-MS: high-performance liquid chromatography mass spectrometry
FPKM: fragments per Kb per million reads
FDR: false discovery rate
DEGs: differentially expressed genes
DAMs: differentially accumulated metabolites
OPLS-DA: orthogonal partial least squares discriminant analysis
VIP: variable importance for the projection
PCA: principal components analysis
GO: gene ontology
KEGG: Kyoto encyclopedia of genes and genomes.
