# Supplementary figures and images for: RNA Interference-Based Genetic Engineering Maize Resistant to Apolygus lucorum Does Not Manifest Unpredictable Unintended Effects Relative to Conventional Breeding: Short Interfering RNA, Transcriptome, and Metabolome Analysis
Source: Front Plant Sci. 2022 Feb 24;13:745708. doi: 10.3389/fpls.2022.745708 (PMC8908210; doi:10.3389/fpls.2022.745708)

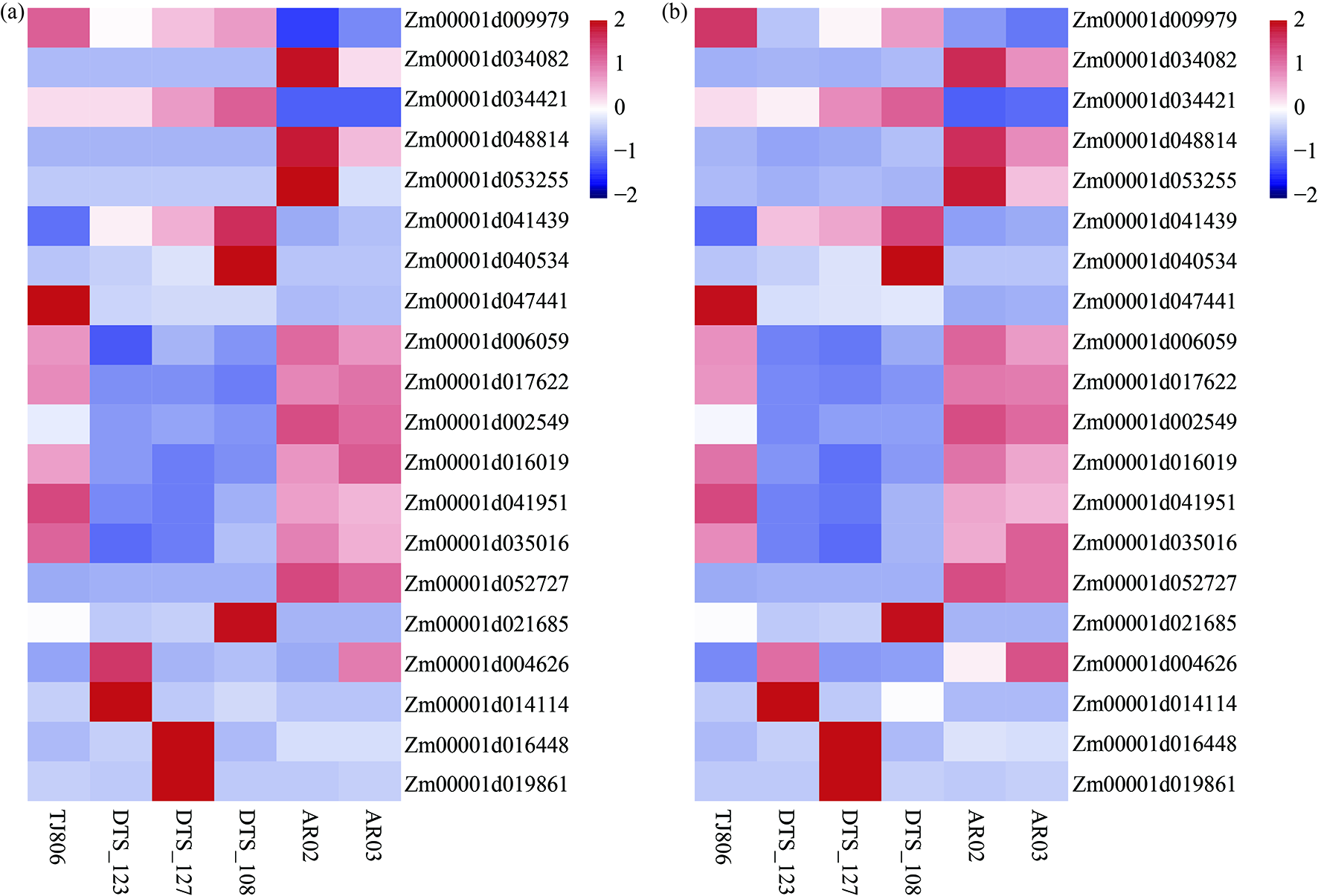

Supplement: Supplementary Figure 1 — Heatmap of verification results of DEGs. (a) Heatmap of verification results of DEGs for RNA-seq data. (b) Heatmap of verification results of DEGs for qPCR data. [file Image_1.TIF]

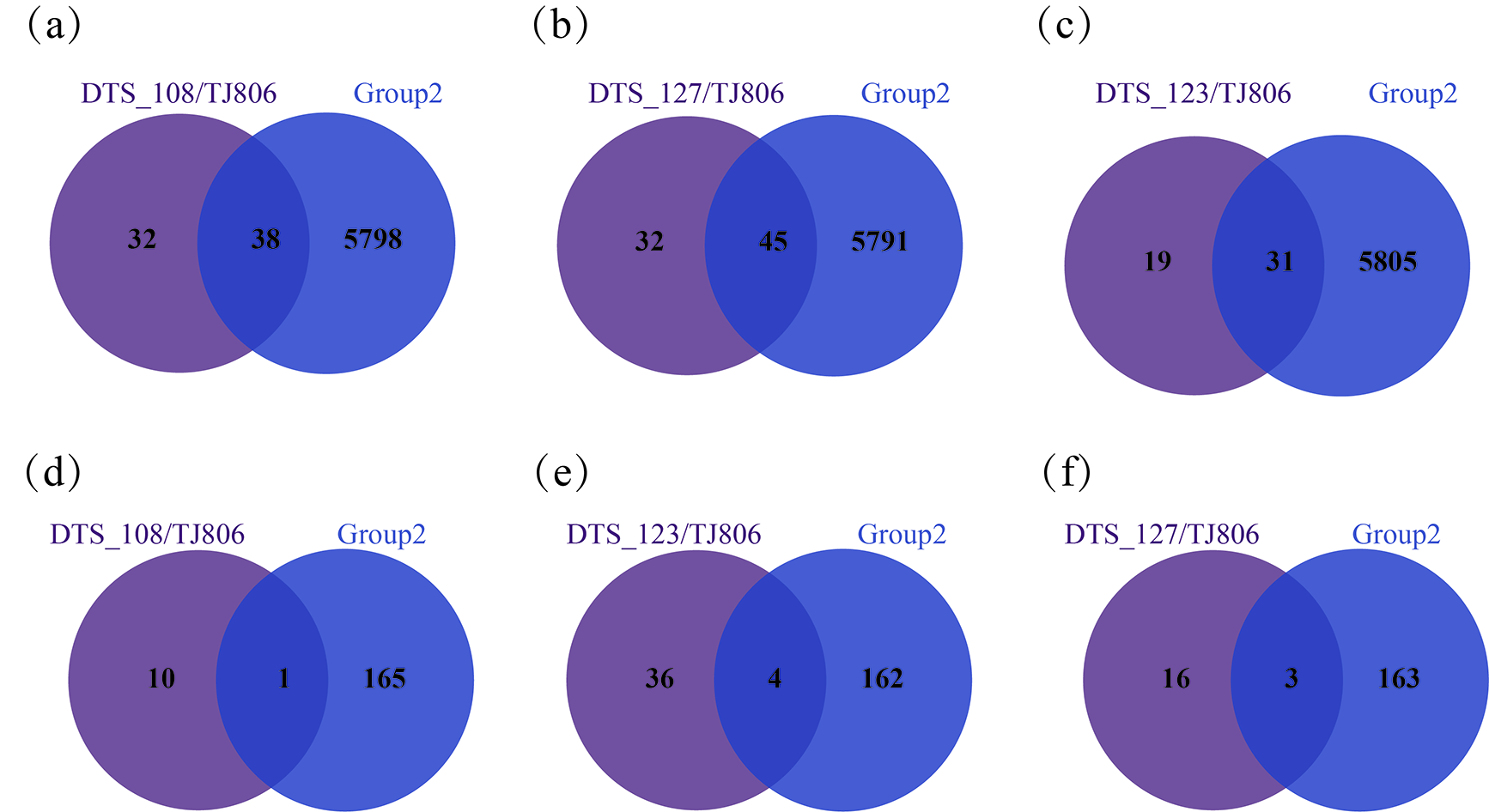

Supplement: Supplementary Figure 2 — Venn diagram analysis between three GE lines and group 2 at the transcriptome and metabolome level. (a–c) Venn diagram analysis of DEGs between three GE lines and group 2 at the transcriptome level. (d–f) Venn diagram analysis of DAMs between three GE lines and group 2 at the metabolome level. [file Image_2.TIF]
