# Supplementary material for: RNA Interference-Based Genetic Engineering Maize Resistant to Apolygus lucorum Does Not Manifest Unpredictable Unintended Effects Relative to Conventional Breeding: Short Interfering RNA, Transcriptome, and Metabolome Analysis
Source: Front Plant Sci. 2022 Feb 24;13:745708. doi: 10.3389/fpls.2022.745708 (PMC8908210; doi:10.3389/fpls.2022.745708)
Supplement: Supplementary file 3 [file Data_Sheet_1.docx]

| Table S1. Maize lines used in this study. | | | | | | |
| --- | --- | --- | --- | --- | --- | --- |
| No. | Maize line | Abbreviation | Insert gene | Promoter | Terminator | Designated in this study |
| 1 | TJ806 | TJ806 | n.a.^1^ | n.a. | n.a. | Parental line |
| 2 | AR02 | AR02 | n.a. | n.a. | n.a. | Cross-breeding line |
| 3 | AR03 | AR03 | n.a. | n.a. | n.a. | Cross-breeding line |
| 4 | DTS_108 | DTS_108 | An inverted repeat of the troponin gene of A. lucorum rLg594(389bp) | pr35S^2^ | tNOS^3^ | Genetic engineering line |
| 5 | DTS_123 | DTS_123 | An inverted repeat of the troponin gene of A. lucorumr Lg594(389bp) | pr35S^2^ | tNOS^3^ | Genetic engineering line |
| 6 | DTS_127 | DTS_127 | An inverted repeat of the troponin gene of A. lucorum rLg594(389bp) | pr35S^2^ | tNOS^3^ | Genetic engineering line |
| Note: 1 n.a. indicated not applicable. 2 Ubiquitin indicated Cauliflower mosaic virus 35S promoter. 3 tNOS is the terminator of nopaline synthase gene. | | | | | | |

| Table S2. Summary of RNA-seq and siRNA mapping results. | | | | |
| --- | --- | --- | --- | --- |
| Sample | Total Raw Reads | Total Clean Reads | Clean Reads Rate (%) | Q30 (%) |
| DTS_108_1 | 15306982 | 12782100 | 83.51 | 96.28 |
| DTS_108_2 | 15223621 | 10279041 | 67.52 | 97.1 |
| DTS_108_3 | 18038997 | 12040393 | 66.75 | 97.27 |
| DTS_123_1 | 21641048 | 12451724 | 57.54 | 95.73 |
| DTS_123_2 | 19736256 | 9974144 | 50.54 | 97.02 |
| DTS_123_3 | 30037959 | 11708289 | 38.98 | 97.17 |
| DTS_127_1 | 24619437 | 13545328 | 55.02 | 97.23 |
| DTS_127_2 | 15135572 | 10426691 | 68.89 | 97.46 |
| DTS_127_3 | 14737346 | 10612239 | 72.01 | 97.27 |
| AR02_1 | 15482489 | 10177778 | 65.74 | 97.39 |
| AR02_2 | 15468113 | 10332674 | 66.8 | 97.5 |
| AR02_3 | 16557830 | 10520654 | 63.54 | 97.25 |
| AR03_1 | 20991929 | 12890144 | 61.41 | 97.48 |
| AR03_2 | 18752120 | 11245376 | 59.97 | 97.47 |
| AR03_3 | 19389654 | 11220074 | 57.87 | 97.27 |
| TJ806_1 | 25709328 | 14050691 | 54.65 | 97.43 |
| TJ806_2 | 16349085 | 10706049 | 65.48 | 97.31 |
| TJ806_3 | 17669909 | 11189041 | 63.32 | 96.86 |

| Table S3. Summary of RNA-seq and transcriptome mapping results. | | | | | | | |
| --- | --- | --- | --- | --- | --- | --- | --- |
| Sample | Raw Reads | Clean Reads | Raw Base | Clean Base | Effective Rate(%) | Q30(%) | Percentage of reads with gene coverage of 90%-100%(%) |
| DTS-108-1 | 56,860,076 | 54,529,166 | 8,529,011,400 | 8,179,374,900 | 95.9 | 94.34 | 76.24 |
| DTS-108-2 | 59,863,558 | 56,719,936 | 8,979,533,700 | 8,507,990,400 | 94.75 | 94.11 | 76.25 |
| DTS-108-3 | 62,659,758 | 58,049,610 | 9,398,963,700 | 8,707,441,500 | 92.64 | 94.26 | 76.57 |
| DTS-123-1 | 61,317,882 | 59,116,120 | 9,197,682,300 | 8,867,418,000 | 96.41 | 94.52 | 74.84 |
| DTS-123-2 | 57,383,570 | 55,108,180 | 8,607,535,500 | 8,266,227,000 | 96.03 | 94.5 | 75.78 |
| DTS-123-3 | 53,165,708 | 50,693,906 | 7,974,856,200 | 7,604,085,900 | 95.35 | 94.43 | 75.06 |
| DTS-127-1 | 61,085,014 | 57,626,908 | 9,162,752,100 | 8,644,036,200 | 94.34 | 94.55 | 74.19 |
| DTS-127-2 | 55,930,826 | 53,886,352 | 8,389,623,900 | 8,082,952,800 | 96.34 | 94.4 | 76.36 |
| DTS-127-3 | 54,783,300 | 52,573,760 | 8,217,495,000 | 7,886,064,000 | 95.97 | 94.47 | 75.61 |
| TJ806-1 | 58,834,726 | 56,068,038 | 8,825,208,900 | 8,410,205,700 | 95.3 | 94.38 | 75.54 |
| TJ806-2 | 60,212,976 | 57,682,948 | 9,031,946,400 | 8,652,442,200 | 95.8 | 94.39 | 75.50 |
| TJ806-3 | 56,711,900 | 54,601,714 | 8,506,785,000 | 8,190,257,100 | 96.28 | 94.37 | 76.15 |
| AR02-1 | 53,255,632 | 49,019,188 | 7,988,344,800 | 7,352,878,200 | 92.05 | 94.43 | 69.45 |
| AR02-2 | 63,516,464 | 60,170,158 | 9,527,469,600 | 9,025,523,700 | 94.73 | 94.34 | 76.14 |
| AR02-3 | 54,832,762 | 52,025,988 | 8,224,914,300 | 7,803,898,200 | 94.88 | 94.29 | 74.82 |
| AR03-1 | 54,921,788 | 52,847,266 | 8,238,268,200 | 7,927,089,900 | 96.22 | 94.12 | 75.84 |
| AR03-2 | 57,816,846 | 55,938,078 | 8,672,526,900 | 8,390,711,700 | 96.75 | 94.23 | 76.02 |
| AR03-3 | 48,103,418 | 44,429,056 | 7,215,512,700 | 6,664,358,400 | 92.36 | 94.06 | 75.28 |

| Table S4. Partly metabolic profiles for compounds accumulated in different rice lines. | | | | | | | | | | |
| --- | --- | --- | --- | --- | --- | --- | --- | --- | --- | --- |
| Alignment ID | Compounds | Compounds number | Formula | Ontology | DTS_108 | DTS_123 | DTS_127 | TJ806 | AR02 | AR03 |
| 447 | Triethanolamine | C06771 | C6H15NO3 | 1,2-aminoalcohols | 13299.67 | 16147.33 | 10674.67 | 16577.33 | 13638.00 | 14715.67 |
| 3599 | Fluocinolone Acetonide (Flucort-N) | C02717 | C24H30F2O6 | 21-hydroxysteroids | 1882.67 | 5948.00 | 2152.00 | 4025.67 | 2276.67 | 6059.67 |
| 1437 | Galaxolidone | C00984 | C18H24O2 | 2-benzopyrans | 162692.33 | 61341.67 | 34794.67 | 87987.00 | 66114.33 | 86062.67 |
| 2746 | Kanakugiol | C03840 | C19H20O6 | 2'-Hydroxychalcones | 10618.33 | 1979.67 | 4094.67 | 6325.67 | 3224.33 | 2105.33 |
| 3472 | Propaquizafop | C10172 | C22H22ClN3O5 | 2-phenoxypropionic acid esters | 1963.00 | 4600.33 | 1168.67 | 1943.33 | 3001.33 | 4034.67 |
| 1246 | Melatonin | C01598 | C13H16N2O2 | 3-alkylindoles | 7386.67 | 5210.67 | 3511.00 | 5961.67 | 4784.67 | 4530.00 |
| 301 | Indole-3-Carbinol | C07051 | C9H9NO | 3-alkylindoles | 20408.33 | 9778.67 | 9206.67 | 18740.00 | 7452.67 | 6933.67 |
| 2018 | Malvidin | C08716 | C17H15O7+ | 3'-O-methylated flavonoids | 20725.33 | 35560.33 | 14696.67 | 14852.33 | 29136.33 | 40982.67 |
| 3982 | Tobramycin | C08082 | C18H37N5O9 | 4,6-disubstituted 2-deoxystreptamines | 10254.67 | 10538.00 | 6865.00 | 12706.33 | 7912.33 | 11202.67 |
| 2379 | Byakangelicin | C09141 | C17H18O7 | 5-methoxypsoralens | 28407.33 | 31949.67 | 21006.00 | 41864.00 | 6324.00 | 9063.67 |
| 680 | 6,7-Dihydroxycoumarin | C09263 | C9H6O4 | 6,7-dihydroxycoumarins | 7317.00 | 9187.00 | 8195.00 | 5692.67 | 6459.00 | 5081.67 |
| 446 | 3-Methyladenine | C00913 | C6H7N5 | 6-aminopurines | 16933.67 | 15971.00 | 8627.00 | 12713.33 | 2238.33 | 4733.33 |
| 2006 | Jaceosidin | C10490 | C17H14O7 | 6-O-methylated flavonoids | 15626.67 | 15125.67 | 7289.33 | 31186.67 | 11913.33 | 11779.00 |
| 3269 | Osajin | C00077 | C25H24O5 | 6-prenylated isoflavanones | 576.00 | 527.33 | 397.33 | 775.67 | 476.33 | 1109.00 |
| 849 | Hymecromone | C00530 | C10H8O3 | 7-hydroxycoumarins | 3842.33 | 5142.33 | 3231.33 | 5523.33 | 7328.00 | 11278.33 |
| 544 | Umbelliferone | C09315 | C9H6O3 | 7-hydroxycoumarins | 35152.67 | 40464.00 | 38703.33 | 50185.00 | 47395.00 | 42009.00 |
| 3439 | Irigenin Trimethyl Ether | C07469 | C21H22O8 | 7-O-methylisoflavones | 14344.00 | 12472.67 | 3207.00 | 8621.00 | 5943.00 | 8728.67 |
| 1402 | Isopimpinellin | C07446 | C13H10O5 | 8-methoxypsoralens | 2503.67 | 1415.67 | 485.00 | 1148.67 | 3303.67 | 7904.33 |
| 1374 | Abscisic Acid | C06082 | C15H20O4 | Abscisic acids and derivatives | 3568.33 | 2027.67 | 2333.67 | 2088.33 | 2293.33 | 2171.67 |
| 1205 | N1-Acetylspermine | C02567 | C12H28N4O | Acetamides | 11161.67 | 9323.67 | 4407.67 | 8964.33 | 4312.00 | 6512.33 |
| 3194 | Talatisamine | C09244 | C24H39NO5 | Aconitane-type diterpenoid alkaloids | 4919.67 | 6161.33 | 2838.33 | 5474.67 | 4674.67 | 10393.00 |
| 3066 | Bixin | C08582 | C25H30O4 | Acyclic diterpenoids | 6373.33 | 9171.67 | 4706.33 | 9068.00 | 6185.33 | 6753.00 |
| 515 | Myrcene | C06074 | C10H16 | Acyclic monoterpenoids | 13848.33 | 5280.33 | 10273.67 | 4770.00 | 6568.67 | 14122.00 |
| 609 | Geranic Acid | C11673 | C10H16O2 | Acyclic monoterpenoids | 5136.67 | 3811.00 | 2590.00 | 5098.00 | 2066.33 | 5528.67 |
| 974 | (5E)-6,10-Dimethyl-5,9-Undecadien-2-One | C13297 | C13H22O | Acyclic monoterpenoids | 10820.67 | 9940.00 | 4838.33 | 6185.33 | 4480.67 | 9697.67 |
| 865 | Acetylcarnitine | C02571 | C9H18NO4 | Acyl carnitines | 2195.67 | 1258.33 | 592.00 | 1470.67 | 458.67 | 517.00 |
| 2016 | N-Acetylmuramic Acid | C02713 | C11H19NO8 | Acylaminosugars | 17754.33 | 9427.00 | 8214.33 | 6064.00 | 10128.00 | 30406.67 |
| 75 | Alanine | C00041 | C3H7NO2 | Alanine and derivatives | 4878.67 | 3397.33 | 1448.00 | 2546.00 | 1881.33 | 2657.33 |
| 536 | Alanopine | C03210 | C6H11NO4 | Alanine and derivatives | 30829.33 | 42330.00 | 25598.67 | 30734.00 | 3125.67 | 9739.00 |
| 343 | Trigonelline | C01004 | C7H7NO2 | Alkaloids and derivatives | 497005.00 | 763578.67 | 351565.67 | 660524.00 | 722242.00 | 747728.33 |
| 338 | 2-Hydroxyacetophenone | C07189 | C8H8O2 | Alkyl-phenylketones | 14722.33 | 21549.00 | 15554.00 | 23089.00 | 25124.00 | 24622.33 |
| 1607 | Dyclonine Hydrochloride | C07881 | C18H28ClNO2 | Alkyl-phenylketones | 138062.67 | 160573.67 | 60937.67 | 124139.33 | 62785.33 | 66980.67 |
| 175 | Glycine-Betaine | C00116 | C5H11NO2 | Alpha amino acids | 220837.33 | 535825.00 | 410512.67 | 273547.33 | 210119.00 | 186439.33 |
| 160 | Betaine | C00719 | C5H11NO2 | Alpha amino acids | 1257341.67 | 1373125.33 | 1294834.00 | 1628600.33 | 1367906.00 | 1346048.67 |
| 168 | Betaine | C00719 | C5H11NO2 | Alpha amino acids | 896697.00 | 1068450.33 | 604139.33 | 986989.00 | 1087982.00 | 1273535.67 |
| 328 | Homocysteine | C01586 | C4H9NO2S | Alpha amino acids | 9694.67 | 15451.00 | 4987.00 | 7664.00 | 17430.00 | 21473.67 |
| 298 | Pyroglutamic Acid | C00314 | C5H7NO3 | Alpha amino acids and derivatives | 5859.33 | 6454.67 | 1371.33 | 1844.33 | 1437.00 | 1996.00 |
| 141 | Creatinine | C00791 | C4H7N3O | Alpha amino acids and derivatives | 2136.33 | 2279.33 | 2344.67 | 2078.00 | 1186.67 | 1196.00 |
| 300 | L-Pyroglutamic Acid | C01879 | C5H7NO3 | Alpha amino acids and derivatives | 24113.00 | 16921.33 | 4836.67 | 7727.33 | 4388.33 | 9652.33 |
| 459 | Phenylglycine | C00079 | C8H9NO2 | Amino acids | 28288.00 | 36475.33 | 17062.67 | 26195.33 | 5219.33 | 5079.67 |
| 313 | Leucine | C07905 | C6H13NO2 | Amino acids | 685.67 | 668.33 | 258.67 | 7449.67 | 1309.67 | 4991.00 |
| 1501 | Sulfamethazine | C00042 | C12H14N4O2S | Aminobenzenesulfonamides | 41806.33 | 37041.00 | 35441.00 | 28891.33 | 27317.33 | 26608.33 |
| 1780 | Tobramycin, Ion Source Fragment Bc | C00397 | C12H26N4O5 | Aminocyclitol glycosides | 27319.00 | 16963.67 | 3154.33 | 18637.67 | 841.33 | 5788.00 |
| 2241 | Calycanthine | C10573 | C22H26N4 | Aminoquinolines and derivatives | 5551.67 | 3657.33 | 1681.00 | 3591.00 | 2853.00 | 2899.00 |
| 980 | Prolintane | C00148 | C15H23N | Amphetamines and derivatives | 3547.67 | 6682.33 | 1768.67 | 1694.33 | 730.67 | 715.00 |
| 1581 | 4-Androstene-3,17-Dione | C00280 | C19H26O2 | Androgens and derivatives | 16733.33 | 12575.00 | 7716.67 | 15219.67 | 7383.33 | 11713.67 |
| 1580 | Androstenedione | C00280 | C19H26O2 | Androgens and derivatives | 1784.67 | 642.67 | 2188.67 | 1629.00 | 627.00 | 5459.00 |
| 1632 | Androsterone | C00523 | C19H30O2 | Androgens and derivatives | 289816.67 | 211133.00 | 119576.33 | 340689.00 | 173059.00 | 374911.33 |
| 1424 | Dehydroepiandrosterone | C01227 | C19H28O2 | Androgens and derivatives | 7103.67 | 6374.67 | 2431.00 | 4970.33 | 1807.67 | 2952.33 |
| 3119 | Alfentanil | C08005 | C21H32N6O3 | Anilides | 2380.67 | 3664.33 | 2429.33 | 8983.67 | 7939.33 | 5948.67 |
| 130 | 2-Aminophenol | C01987 | C6H7NO | Aniline and substituted anilines | 19559.33 | 24859.33 | 17644.00 | 31202.67 | 48015.00 | 51787.00 |
| 126 | 4-Aminophenol | C02372 | C6H7NO | Aniline and substituted anilines | 11548.33 | 32162.33 | 23049.33 | 30447.67 | 60076.00 | 59262.33 |
| 128 | 4-Aminophenol | C02372 | C6H7NO | Aniline and substituted anilines | 3730.33 | 13613.33 | 8488.00 | 13922.67 | 62069.00 | 34479.33 |
| 4286 | Carminic Acid | C11254 | C22H20O13 | Anthracenecarboxylic acids | 27353.00 | 24571.33 | 15324.00 | 20254.00 | 13590.00 | 19103.67 |
| 1302 | Danthron | C10312 | C14H8O4 | Anthraquinones | 2746.67 | 3807.67 | 2250.33 | 5102.00 | 6057.33 | 5241.00 |
| 1419 | Leflunomide | C00318 | C12H9F3N2O2 | Aromatic anilides | 365.33 | 1372.33 | 421.67 | 703.67 | 1694.67 | 1314.00 |
| 2700 | Galbulin | C08526 | C22H28O4 | Aryltetralin lignans | 2408.67 | 3352.00 | 3398.33 | 7042.33 | 2933.00 | 7885.67 |
| 634 | N-Acetyl-L-Aspartic Acid | C01042 | C6H9NO5 | Aspartic acid and derivatives | 8383.33 | 13393.00 | 6916.33 | 12277.00 | 9358.67 | 12236.00 |
| 2891 | Sulfasalazine (Azulfidine) | C19530 | C18H14N4O5S | Azobenzenes | 6090.67 | 4120.00 | 1703.33 | 4620.00 | 268.33 | 344.00 |
| 1353 | Methohexital | C07844 | C14H18N2O3 | Barbituric acid derivatives | 4277.67 | 7729.00 | 2016.00 | 5071.33 | 7571.33 | 9332.00 |
| 204 | Benzamide | C09815 | C7H7NO | Benzamides | 7037.33 | 11315.67 | 4121.00 | 7735.67 | 8475.33 | 15057.00 |
| 1673 | N4-Acetylsulfamethoxazole | C13061 | C12H13N3O4S | Benzenesulfonamides | 88275.67 | 41354.33 | 13503.33 | 35703.00 | 27523.33 | 77302.67 |
| 414 | 5,6-Dimethylbenzimidazole | C03114 | C9H10N2 | Benzimidazoles | 9909.67 | 7665.67 | 4764.33 | 9732.67 | 574.00 | 7936.00 |
| 971 | Euparin | C00468 | C13H12O3 | Benzofurans | 3132.67 | 2226.33 | 1204.00 | 2833.33 | 2311.00 | 3352.67 |
| 1518 | Dibutyl Phthalate | C14214 | C16H22O4 | Benzoic acid esters | 26251.67 | 28764.00 | 28607.00 | 27919.67 | 33722.00 | 30006.33 |
| 577 | Phthalamic Acid | C00588 | C8H7NO3 | Benzoic acids | 259264.33 | 229031.33 | 187866.67 | 282589.33 | 186191.00 | 417313.00 |
| 2415 | Ochropposinine | C08775 | C21H30N2O3 | Beta carbolines | 7107.00 | 6088.67 | 2502.33 | 6435.00 | 4331.00 | 4261.33 |
| 3293 | Irbesartan (Avapro) | C19837 | C25H28N6O | Biphenyls and derivatives | 46306.00 | 31278.67 | 42297.00 | 75537.67 | 51766.33 | 47479.67 |
| 4174 | Brassinolide | C11049 | C28H48O6 | Brassinolides and derivatives | 30139.00 | 49128.33 | 16294.00 | 33322.33 | 14494.67 | 30354.33 |
| 806 | Ascorbic Acid | C01041 | C6H8O6 | Butenolides | 42143.67 | 67914.00 | 37434.00 | 67554.67 | 69467.33 | 84766.33 |
| 699 | Caffeic Acid | C01481 | C9H8O4 | Caffeic acid and derivatives | 8778.67 | 8474.67 | 2888.33 | 11668.00 | 4891.67 | 7903.33 |
| 4609 | Lanciferine | C01717 | C31H32N2O7 | Carbazoles | 8752.00 | 5666.67 | 3658.67 | 6996.00 | 6835.67 | 9874.00 |
| 3485 | Fusaproliferin_130137 | C00122 | C27H40O5 | Carboxylic acid esters | 7172.00 | 18970.67 | 6481.00 | 9281.00 | 6015.00 | 8384.67 |
| 5970 | Digoxin | C06956 | C41H64O14 | Cardenolide glycosides and derivatives | 1435.33 | 1261.00 | 979.00 | 1914.67 | 2124.67 | 2943.00 |
| 3461 | Strophanthidin | C03470 | C23H32O6 | Cardenolides and derivatives | 5201.00 | 3907.33 | 5421.00 | 4780.33 | 4904.67 | 5075.33 |
| 539 | Carnitine | C00318 | C7H15NO3 | Carnitines | 31245.67 | 9120.33 | 5749.33 | 7987.33 | 3901.00 | 3563.67 |
| 1839 | Catechin | C06562 | C15H14O6 | Catechins | 6464.67 | 7776.67 | 6284.33 | 7763.33 | 6560.67 | 2896.67 |
| 483 | Dopamine | C03758 | C8H11NO2 | Catecholamines and derivatives | 21178.33 | 40402.67 | 17782.00 | 24552.67 | 1422.67 | 5717.00 |
| 3043 | Cholesterol | C00187 | C27H46O | Cholesterols and derivatives | 3894.67 | 4246.67 | 2533.00 | 4466.33 | 3691.33 | 1612.33 |
| 119 | Choline | C00114 | C5H14NO | Cholines | 51035.33 | 62390.00 | 30102.67 | 61784.33 | 55772.67 | 53788.00 |
| 120 | Choline | C00114 | C5H14NO | Cholines | 1997522.33 | 1921309.33 | 1216402.33 | 2129198.67 | 1866007.33 | 2145034.00 |
| 909 | Ethyl Caffeate | C05141 | C11H12O4 | Coumaric acids and derivatives | 5273.67 | 5408.33 | 2991.00 | 5463.00 | 2626.33 | 2631.67 |
| 2478 | Aesculin | C09264 | C15H16O9 | Coumarin glycosides | 13342.00 | 15597.33 | 11261.33 | 18367.00 | 9584.00 | 15054.33 |
| 1478 | Ethyl 2-((4-Methyl-2-Oxo-2H-Chromen-7-Yl)Oxy)Propanoate | C09263 | C15H16O5 | Coumarins and derivatives | 4810.33 | 7230.67 | 4080.67 | 7015.00 | 5976.33 | 6066.33 |
| 4544 | Cucurbitacin D | C08796 | C30H44O7 | Cucurbitacins | 39525.00 | 53626.33 | 31437.67 | 55291.67 | 26675.33 | 24549.67 |
| 2613 | Tetrahydrocurcumin | C00535 | C21H24O6 | Curcuminoids | 4483.00 | 2946.33 | 1169.67 | 4202.00 | 2393.67 | 10036.00 |
| 2777 | Curcumin | C10443 | C21H20O6 | Curcuminoids | 7711.00 | 8577.67 | 5068.00 | 11942.67 | 8953.67 | 3698.67 |
| 1413 | Linamarin | C01594 | C10H17NO6 | Cyanogenic glycosides | 15863.00 | 9132.00 | 6404.33 | 19682.00 | 11445.33 | 13524.67 |
| 968 | Quebrachitol | C00396 | C7H14O6 | Cyclohexanols | 8625.33 | 9859.33 | 6591.00 | 13262.33 | 13345.33 | 15782.33 |
| 108 | Cyclohexanamine | C00571 | C6H13N | Cyclohexylamines | 8700.33 | 9369.67 | 4893.67 | 8875.00 | 5751.67 | 8067.33 |
| 107 | Cyclohexylamine | C00571 | C6H13N | Cyclohexylamines | 643.33 | 4297.67 | 584.67 | 4380.67 | 1586.67 | 1946.67 |
| 203 | Cysteine | C00097 | C3H7NO2S | Cysteine and derivatives | 6945.67 | 4236.00 | 3812.67 | 5769.00 | 5067.33 | 5723.33 |
| 3140 | Simvastatin | C00493 | C25H38O5 | Delta valerolactones | 23264.00 | 8687.00 | 8836.33 | 13835.00 | 16935.33 | 25185.67 |
| 2980 | Lovastatin | C07074 | C24H36O5 | Delta valerolactones | 81585.67 | 106594.00 | 58128.33 | 92981.00 | 32733.33 | 42248.00 |
| 2871 | Protocetraric Acid | C07406 | C18H14O9 | Depsides and depsidones | 8626.33 | 61626.00 | 7687.33 | 12538.00 | 26963.67 | 32868.33 |
| 1139 | Pesticide1_Dicrotophos_C8H16No5P_(2E)-4-(Dimethylamino)-4-Oxo-2-Buten-2-Yl Dimethyl Phosphate | C07706 | C8H16NO5P | Dialkyl phosphates | 1903.33 | 1260.00 | 1441.00 | 4802.67 | 940.33 | 738.67 |
| 1148 | Pirimicarb | C10166 | C11H18N4O2 | Dialkylarylamines | 30159.33 | 53550.00 | 18226.67 | 19226.00 | 11877.00 | 33044.00 |
| 2617 | Arctigenin | C10545 | C21H24O6 | Dibenzylbutyrolactone lignans | 63145.00 | 8520.67 | 3046.00 | 11882.67 | 1289.67 | 34992.67 |
| 359 | Fumaric Acid | C17854 | C4H4O4 | Dicarboxylic acids and derivatives | 4495.33 | 4775.67 | 2182.67 | 5226.33 | 2216.33 | 2803.67 |
| 1990 | Aflatoxin G1 | C16755 | C17H12O7 | Difurocoumarolactones | 7840.00 | 7543.00 | 4674.67 | 7775.33 | 3887.00 | 4653.33 |
| 2369 | Dihydrosanguinarine | C05191 | C20H15NO4 | Dihydrobenzophenanthridine alkaloids | 10569.67 | 12567.67 | 7663.33 | 16679.67 | 10270.00 | 14633.33 |
| 3321 | Deoxycholic Acid | C04483 | C24H40O4 | Dihydroxy bile acids, alcohols and derivatives | 73361.33 | 54409.67 | 67858.33 | 120835.67 | 96353.33 | 108933.00 |
| 2671 | Topiramate | C10827 | C12H21NO8S | Dioxolopyrans | 7629.67 | 9100.67 | 6394.67 | 9314.67 | 6233.67 | 8572.33 |
| 529 | Ala-Ala | C00993 | C6H12N2O3 | Dipeptides | 7576.33 | 6499.67 | 3152.67 | 6128.33 | 2257.33 | 4621.00 |
| 1973 | Tolterodine | C07949 | C22H31NO | Diphenylmethanes | 31581.67 | 57250.00 | 4852.67 | 7957.33 | 7262.00 | 19517.67 |
| 4734 | Atorvastatin | C06834 | C33H35FN2O5 | Diphenylpyrroles | 33042.00 | 42455.33 | 38642.67 | 69609.33 | 32874.67 | 54299.67 |
| 6141 | Tolytoxin | C07750 | C46H75NO13 | Diterpene lactones | 59313.67 | 63122.67 | 66027.00 | 85568.00 | 82413.33 | 136638.67 |
| 4038 | Cephaeline | C09390 | C28H38N2O4 | Emetine alkaloids | 4810.00 | 5130.33 | 3012.00 | 5102.33 | 2281.33 | 3656.33 |
| 2758 | Campesterol | C01789 | C28H48O | Ergosterols and derivatives | 5016.00 | 10919.67 | 8217.00 | 8438.67 | 12057.67 | 15941.67 |
| 1998 | Trilostane | C12580 | C20H27NO3 | Estrane steroids | 4585.00 | 2290.33 | 1127.67 | 2019.33 | 756.00 | 1395.33 |
| 1283 | Alpha-Estradiol | C02537 | C18H24O2 | Estrogens and derivatives | 7909.33 | 5755.33 | 3102.67 | 7478.67 | 2748.00 | 5831.67 |
| 1595 | (16alpha,17beta)-Estra-1,3,5(10)-Triene-3,16,17-Triol | C05141 | C18H24O3 | Estrogens and derivatives | 53238.67 | 34143.00 | 19676.33 | 44255.00 | 26745.00 | 58305.33 |
| 1721 | 2-Methoxyestradiol | C05302 | C19H26O3 | Estrogens and derivatives | 13912.67 | 13036.00 | 5601.00 | 11020.00 | 7053.67 | 19111.00 |
| 1464 | Nandrolone | C07254 | C18H26O2 | Estrogens and derivatives | 20719.67 | 11064.00 | 5273.00 | 10185.33 | 7833.33 | 13658.67 |
| 1695 | Norethindrone (Norethisterone) | C10112 | C20H26O2 | Estrogens and derivatives | 20056.67 | 18080.67 | 9776.33 | 14997.00 | 7852.33 | 11281.00 |
| 1683 | Rosin | C22165 | C15H20O6 | Fatty acyl glycosides of mono- and disaccharides | 3473.67 | 6124.67 | 2141.00 | 4011.00 | 4220.00 | 6787.67 |
| 1736 | Taxifolin | C02592 | C15H12O7 | Flavanonols | 1563.00 | 8682.00 | 1657.67 | 2751.67 | 4479.67 | 6692.33 |
| 3089 | (-)-Riboflavin | C00255 | C17H20N4O6 | Flavins | 1516.33 | 1276.33 | 1846.33 | 2062.00 | 1621.33 | 584.67 |
| 3133 | Liquiritin | C16989 | C21H22O9 | Flavonoid O-glycosides | 963.67 | 1316.00 | 894.33 | 1552.00 | 2528.00 | 5163.33 |
| 3677 | Quercetin 3-O-Alpha-L-Arabinopyranoside | C01879 | C20H18O11 | Flavonoid-3-O-glycosides | 303.33 | 2542.67 | 166.33 | 1073.00 | 601.33 | 1956.00 |
| 3644 | Afzelin | C16911 | C21H20O10 | Flavonoid-3-O-glycosides | 8184.33 | 9110.67 | 2957.33 | 7809.00 | 10881.33 | 10207.67 |
| 5637 | Icariin | C00262 | C33H40O15 | Flavonoid-7-O-glycosides | 12791.00 | 19786.67 | 11927.00 | 19743.00 | 11631.33 | 17770.00 |
| 1919 | Myricetin | C10107 | C15H10O8 | Flavonols | 5206.00 | 4967.33 | 3557.00 | 6246.33 | 3324.33 | 1670.00 |
| 702 | 9-Fluorenone | C06712 | C13H8O | Fluorenes | 2251.67 | 2219.33 | 1969.67 | 2192.67 | 8470.67 | 9772.33 |
| 2351 | L-Asarinin | C01594 | C20H18O6 | Furanoid lignans | 19803.00 | 24090.00 | 17699.00 | 25986.67 | 25873.67 | 24901.00 |
| 2026 | Picrotin | C10871 | C15H18O7 | Furopyrans | 43644.67 | 35822.67 | 17210.67 | 31771.00 | 21896.33 | 35879.67 |
| 955 | Propyl Gallate | C00163 | C10H12O5 | Galloyl esters | 7010.00 | 1572.00 | 3215.00 | 13881.00 | 3519.00 | 1579.00 |
| 411 | 4-Guanidinobutyric Acid | C01035 | C5H11N3O2 | Gamma amino acids and derivatives | 7793.00 | 5430.00 | 3368.00 | 6104.00 | 4203.67 | 5241.67 |
| 410 | 4-Acetamidobutanoate | C02946 | C6H11NO3 | Gamma amino acids and derivatives | 6640.00 | 5465.67 | 3445.33 | 6954.00 | 8650.00 | 7755.33 |
| 1579 | Parthenolide | C00864 | C15H20O3 | Germacranolides and derivatives | 2732.33 | 7393.00 | 976.00 | 2191.00 | 9123.67 | 8872.33 |
| 2301 | 10-Gingerol | C10462 | C21H34O4 | Gingerols | 7859.00 | 8671.33 | 2365.00 | 8601.00 | 3520.67 | 4097.67 |
| 2766 | Megestrol Acetate | C07120 | C24H32O4 | Gluco/mineralocorticoids, progestogins and derivatives | 11177.00 | 10000.00 | 5811.00 | 11760.67 | 7257.00 | 6024.00 |
| 4583 | Mometasone Furoate | C07816 | C27H30Cl2O6 | Gluco/mineralocorticoids, progestogins and derivatives | 45820.67 | 13215.33 | 5668.00 | 11103.00 | 2302.33 | 5289.00 |
| 621 | Sn-Glycerol 3-Phosphate | C00670 | C3H9O6P | Glycerophosphates | 3508.00 | 2378.67 | 979.00 | 2263.33 | 2146.00 | 2234.67 |
| 1294 | Isocurcumenol | C02632 | C15H22O2 | Guaianes | 10699.00 | 11310.67 | 6751.00 | 13280.00 | 12017.33 | 11025.00 |
| 1112 | Curcumenol | C16942 | C15H22O2 | Guaianes | 18204.00 | 8296.00 | 9892.67 | 11375.00 | 8503.00 | 14830.67 |
| 690 | Hippuric Acid | C01709 | C9H9NO3 | Hippuric acids | 12359.67 | 13563.67 | 2830.67 | 10655.00 | 8596.00 | 7978.33 |
| 2589 | Rebamipide | C11372 | C19H15ClN2O4 | Hippuric acids | 6432.00 | 5323.67 | 7232.33 | 5365.67 | 6515.00 | 4095.33 |
| 4694 | Gomisin D | C15557 | C28H34O10 | Hydrolyzable tannins | 2043.33 | 3421.00 | 501.33 | 1095.00 | 362.33 | 689.00 |
| 134 | Hydroquinone | C13534 | C6H6O2 | Hydroquinones | 59883.00 | 123483.00 | 15248.33 | 38303.33 | 32585.00 | 32990.33 |
| 692 | Meglutol | C03761 | C6H10O5 | Hydroxy fatty acids | 19972.67 | 18100.67 | 10808.67 | 17694.00 | 11145.00 | 15588.33 |
| 698 | Caffeic Acid | C01481 | C9H8O4 | Hydroxycinnamic acids | 2752.33 | 1409.00 | 927.00 | 1440.67 | 7915.33 | 5866.00 |
| 2092 | Eicosanoids_18-Hepe_C20H30O3 | C06231 | C20H30O3 | Hydroxyeicosapentaenoic acids | 376927.33 | 343838.33 | 195141.00 | 389256.67 | 304641.00 | 356003.33 |
| 2570 | Glycycoumarin | C01921 | C21H20O6 | Hydroxyisoflavonoids | 37591.67 | 45694.33 | 23298.33 | 43549.33 | 25608.67 | 32689.33 |
| 291 | 5-Methylcytosine | C02376 | C5H7N3O | Hydroxypyrimidines | 8010.67 | 7551.00 | 5095.67 | 8886.00 | 5885.67 | 6865.00 |
| 696 | Allantoin | C01551 | C4H6N4O3 | Imidazoles | 12118.33 | 319511.33 | 7114.67 | 169122.67 | 100927.33 | 239611.00 |
| 369 | Urocanate | C00785 | C6H6N2O2 | Imidazolyl carboxylic acids and derivatives | 27771.00 | 30181.33 | 15977.33 | 26780.00 | 27323.67 | 27427.67 |
| 370 | Urocanic Acid | C00785 | C6H6N2O2 | Imidazolyl carboxylic acids and derivatives | 16425.67 | 17431.00 | 21659.33 | 11420.33 | 8923.67 | 10016.67 |
| 637 | 3-Indoleacetic Acid | C00954 | C10H9NO2 | Indole-3-acetic acid derivatives | 6616.33 | 2283.00 | 3237.33 | 3773.67 | 2962.67 | 7203.33 |
| 534 | Indole-3-Carboxylic Acid | C02693 | C9H7NO2 | Indolecarboxylic acids and derivatives | 5743.33 | 7146.67 | 3507.33 | 9363.67 | 11416.33 | 8344.67 |
| 533 | 1H-Indole-3-Carboxylic Acid | C19837 | C9H7NO2 | Indolecarboxylic acids and derivatives | 22985.00 | 16451.33 | 21160.33 | 17567.67 | 27890.00 | 46571.67 |
| 2781 | Harpagide | C00144 | C15H24O10 | Iridoid O-glycosides | 14846.00 | 15620.33 | 10153.33 | 12248.67 | 9841.67 | 12633.33 |
| 4318 | Harpagoside | C08305 | C24H30O11 | Iridoid O-glycosides | 3371.33 | 7972.67 | 5504.33 | 9107.00 | 6860.33 | 6870.00 |
| 2568 | Ethyl 3-(2,3-Dihydrobenzo[b][1,4]Dioxin-6-Yl)-7-Hydroxy-4-Oxo-4H-Chromene-2-Carboxylate | C09264 | C20H16O7 | Isoflavones | 5014.00 | 7014.67 | 2958.67 | 5694.00 | 8124.67 | 16933.33 |
| 1038 | Methyl Jasmonate | C11512 | C13H20O3 | Jasmonic acids | 62619.00 | 61309.67 | 45162.33 | 51131.33 | 22278.67 | 21075.00 |
| 1475 | Dihydromethysticin | C09926 | C15H16O5 | Kavalactones | 664.67 | 5243.67 | 1365.33 | 832.67 | 5223.67 | 1136.00 |
| 1939 | Pseudo-Anisatin | C08907 | C15H22O6 | Lactones | 11930.67 | 13467.00 | 11673.00 | 18927.67 | 7108.00 | 18019.33 |
| 661 | Alliin | C08265 | C6H11NO3S | L-alpha-amino acids | 14954.33 | 17964.67 | 6316.33 | 18249.00 | 7998.00 | 7004.67 |
| 312 | Leucine | C00123 | C6H13NO2 | Leucine and derivatives | 17971.33 | 15386.33 | 10253.00 | 23015.33 | 22020.67 | 18854.67 |
| 4658 | Tracheloside | C07502 | C27H34O12 | Lignan glycosides | 4316.67 | 2618.33 | 2055.33 | 2714.67 | 229.33 | 297.67 |
| 5282 | Thalsimidine | C07910 | C37H38N2O7 | Lignols | 494.33 | 1471.00 | 672.33 | 988.67 | 990.00 | 3268.33 |
| 3907 | Obacunone | C01933 | C26H30O7 | Limonoids | 3009.00 | 3657.00 | 3427.00 | 4549.33 | 11482.33 | 24550.67 |
| 1722 | Linoleic Acid | C01595 | C18H32O2 | Lineolic acids and derivatives | 5416.00 | 5754.00 | 1951.67 | 7867.00 | 2431.67 | 4869.33 |
| 1523 | Linolenic Acid | C06427 | C18H30O2 | Lineolic acids and derivatives | 22686.33 | 37385.00 | 17179.67 | 25674.00 | 7227.67 | 8074.67 |
| 2554 | Icos-19-Ene-1,2,4-Triol | C01588 | C20H40O3 | Long-chain fatty alcohols | 24205.00 | 22328.33 | 12874.33 | 24223.00 | 19012.67 | 20124.67 |
| 1238 | Sophoridine | C10822 | C15H24N2O | Matrine alkaloids | 4037.33 | 17229.33 | 484.00 | 1587.67 | 219.67 | 505.00 |
| 937 | Azelaic Acid | C08261 | C9H16O4 | Medium-chain fatty acids | 5102.00 | 4083.33 | 3395.00 | 5329.67 | 2203.00 | 3276.67 |
| 516 | d-Limonene | C06099 | C10H16 | Menthane monoterpenoids | 14867.00 | 9722.00 | 5601.00 | 7500.33 | 5997.00 | 13080.00 |
| 444 | Methionine | C00073 | C5H11NO2S | Methionine and derivatives | 10683.33 | 10125.67 | 4461.33 | 9340.33 | 3860.33 | 4729.33 |
| 912 | Sinapoyl Aldehyde | C00482 | C11H12O4 | Methoxyphenols | 14762.00 | 21510.00 | 11242.67 | 17167.33 | 11024.67 | 12305.33 |
| 463 | Vanillin | C00755 | C8H8O3 | Methoxyphenols | 62760.00 | 263626.00 | 47742.00 | 42701.33 | 165907.00 | 149113.67 |
| 1087 | Sinapyl Alcohol | C05610 | C11H14O4 | Methoxyphenols | 11634.33 | 11603.00 | 7617.67 | 10763.00 | 7738.00 | 10245.00 |
| 1755 | Capsaicin | C06866 | C18H27NO3 | Methoxyphenols | 8146.00 | 3409.33 | 1399.33 | 2621.67 | 2102.33 | 12807.67 |
| 451 | 2-Methoxy-4-Vinylphenol | C17883 | C9H10O2 | Methoxyphenols | 7590.33 | 9444.67 | 5626.33 | 7488.33 | 4625.67 | 6458.00 |
| 608 | Vanillic Acid | C06672 | C8H8O4 | M-methoxybenzoic acids and derivatives | 39170.67 | 26262.67 | 17658.33 | 27191.67 | 70167.67 | 196798.00 |
| 111 | 1-Hexylamine | C08306 | C6H15N | Monoalkylamines | 59073.33 | 65034.00 | 62431.67 | 59785.67 | 55370.00 | 67909.00 |
| 2312 | Sinomenine | C02325 | C19H23NO4 | Morphinans | 7997.67 | 2500.67 | 4676.67 | 6191.33 | 4024.00 | 872.00 |
| 879 | Panthenol | C00249 | C9H19NO4 | N-acyl amines | 5676.67 | 4725.33 | 3921.33 | 5578.00 | 4056.33 | 7972.67 |
| 1660 | Tbuo-Val-Hexanoate (Tert-Butyl Hexanoylvalinate) | C01617 | C15H29NO3 | N-acyl-alpha amino acids and derivatives | 12219.67 | 14423.67 | 7161.00 | 13917.33 | 17505.67 | 22907.00 |
| 558 | N-Acetylcysteine | C06809 | C5H9NO3S | N-acyl-L-alpha-amino acids | 11043.33 | 13240.33 | 6036.00 | 16218.33 | 14674.67 | 21624.67 |
| 216 | Niacinamide | C09871 | C6H6N2O | Nicotinamides | 59754.33 | 32496.67 | 29980.67 | 49859.67 | 125473.67 | 140724.00 |
| 2418 | Isomaltulose | C00407 | C12H22O11 | O-glycosyl compounds | 466093.67 | 462364.00 | 249815.67 | 400494.33 | 242328.33 | 326095.67 |
| 4412 | Maltotriose | C01835 | C18H32O16 | Oligosaccharides | 2843.00 | 204.67 | 127.67 | 215.00 | 159.33 | 156.00 |
| 4411 | Melezitose | C08243 | C18H32O16 | Oligosaccharides | 37591.00 | 10650.00 | 4217.00 | 9337.67 | 1942.33 | 4265.33 |
| 330 | Adenine | C00147 | C5H5N5 | Others | 395614.00 | 341272.00 | 249963.00 | 425153.33 | 310641.67 | 395071.33 |
| 55 | Pyrimidine | C00250 | C4H4N2 | Others | 22073.67 | 11877.33 | 31539.67 | 46159.00 | 57362.00 | 39029.33 |
| 6258 | Rifapentine | C00255 | C47H64N4O12 | Others | 3296.33 | 5861.67 | 5470.00 | 6282.33 | 6599.00 | 5153.67 |
| 2856 | Thiophanic Acid | C00378 | C14H6Cl4O5 | Others | 16690.00 | 22822.67 | 15769.00 | 28905.67 | 17646.00 | 25071.00 |
| 326 | Carveol | C00964 | C10H16O | Others | 9227.33 | 10229.33 | 5259.00 | 9067.33 | 5399.33 | 7492.00 |
| 3843 | 18b-Glycyrrhetinic Acid | C02283 | C30H46O4 | Others | 913.67 | 3239.67 | 1357.67 | 827.33 | 4043.67 | 3849.67 |
| 1788 | Zolpidem Tartrate | C07219 | C19H21N3O | Others | 4606.67 | 5069.67 | 2595.67 | 5118.33 | 4110.00 | 3196.67 |
| 2396 | Nalbuphine | C07251 | C21H27NO4 | Others | 2058.33 | 35223.33 | 708.33 | 6087.67 | 7346.00 | 23926.67 |
| 4773 | Phenylalanine Conjugated Chenodeoxycholic Acid | C07432 | C33H49NO5 | Others | 18918.33 | 13585.67 | 17401.00 | 17568.33 | 17026.67 | 16386.00 |
| 3601 | Diphenoxylate | C07872 | C30H32N2O2 | Others | 2162.00 | 5728.67 | 1190.00 | 3284.00 | 3791.00 | 4844.67 |
| 3595 | Roccellin | C08059 | C21H24O11 | Others | 1058.33 | 2946.00 | 892.67 | 3394.33 | 1306.67 | 4224.33 |
| 4921 | Putative Tryptophane Conjugated Chenodeoxycholic Acid | C10523 | C35H50N2O5 | Others | 10504.33 | 11069.00 | 3955.00 | 4808.67 | 3492.00 | 4586.67 |
| 2867 | Saliniketal A | C11770 | C22H37NO5 | Others | 4353.00 | 6151.67 | 1883.00 | 4251.00 | 4218.33 | 6833.67 |
| 3528 | Glycohyocholic Acid | C13044 | C26H43NO6 | Others | 100269.00 | 129442.00 | 45540.33 | 151452.00 | 205117.67 | 54554.67 |
| 742 | 2,6-Dimethoxyquinone | C10331 | C8H8O4 | P-benzoquinones | 14824.00 | 20580.33 | 13942.67 | 27371.33 | 21873.67 | 28323.33 |
| 1201 | N-L-Leucyl-L-Leucine | C00153 | C12H24N2O3 | Peptides | 12853.67 | 18912.67 | 14028.67 | 25049.67 | 16192.33 | 25354.33 |
| 4200 | Strepin P1 | C05442 | C25H40N6O5 | Peptides | 3989.00 | 7156.00 | 2444.67 | 5392.67 | 4400.33 | 2259.33 |
| 333 | 2-Phenylacetamide | C02505 | C8H9NO | Phenylacetamides | 30925.67 | 34327.67 | 19364.00 | 23603.33 | 5805.00 | 7958.33 |
| 587 | Phenylalanine | C05332 | C9H11NO2 | Phenylalanine and derivatives | 5533.67 | 6253.67 | 2208.33 | 2959.33 | 1577.33 | 1450.33 |
| 1259 | n-Methyl-2,4-Dihydroxy-3-Phenylquinoline | C00153 | C16H13NO2 | Phenylquinolines | 33597.33 | 19454.00 | 13971.00 | 17372.00 | 8223.33 | 23635.67 |
| 2408 | Mycophenolic Acid | C20380 | C17H20O6 | Phthalides | 4118.33 | 3860.00 | 1539.67 | 3106.00 | 3065.67 | 6264.33 |
| 1217 | Haematommic Acid, Ethyl Ester | C00387 | C11H12O5 | p-Hydroxybenzoic acid alkyl esters | 27591.67 | 19239.00 | 8544.67 | 21675.67 | 15937.67 | 21156.00 |
| 899 | Miglitol | C07708 | C8H17NO5 | Piperidines | 13162.33 | 10200.33 | 3412.33 | 10088.00 | 8793.67 | 11705.00 |
| 4087 | Mls001333570-01!55297-96-6 | - | C32H51NO8S | Pleuromutilin and derivatives | 6774.00 | 5946.33 | 2508.00 | 6174.67 | 2567.00 | 5347.33 |
| 2413 | Tabersonine | C01533 | C21H24N2O2 | Plumeran-type alkaloids | 4751.67 | 2438.67 | 3687.67 | 5137.00 | 2626.00 | 2597.67 |
| 3400 | Podophyllotoxin | C10874 | C22H22O8 | Podophyllotoxins | 48652.33 | 48507.00 | 26738.67 | 60844.67 | 42987.67 | 108842.67 |
| 1554 | Hexaethylene Glycol | - | C12H26O7 | Polyethylene glycols | 35842.67 | 55054.00 | 54711.67 | 53100.67 | 26113.33 | 26311.00 |
| 5538 | Substance P | C19506 | C63H98N18O13S | Polypeptides | 6007.33 | 13417.67 | 4718.33 | 9348.00 | 1760.00 | 1810.33 |
| 401 | Proline Betaine | C00410 | C7H13NO2 | Proline and derivatives | 4479.00 | 6442.67 | 3303.33 | 5631.67 | 2838.00 | 14441.67 |
| 3242 | Clindamycin | C06914 | C18H33ClN2O5S | Proline and derivatives | 11384.33 | 13841.67 | 8084.33 | 10693.33 | 12434.67 | 9724.33 |
| 2352 | Hydroprotopine | C00735 | C20H20NO5+ | Protoberberine alkaloids and derivatives | 6278.33 | 3205.00 | 6282.00 | 12753.00 | 1904.33 | 1681.00 |
| 2410 | Epiberberine | C10343 | C20H18NO4+ | Protoberberine alkaloids and derivatives | 11076.00 | 7290.33 | 3594.00 | 8725.67 | 866.67 | 1141.67 |
| 2293 | Isopropylidenylacetyl-Marmesin | C02162 | C19H20O5 | Psoralens | 1354.67 | 939.67 | 293.00 | 704.00 | 567.67 | 691.67 |
| 1397 | Adenosine | C00212 | C10H13N5O4 | Purine nucleosides | 5489272.00 | 4465922.33 | 3110153.00 | 5510489.00 | 3863496.67 | 5126396.33 |
| 2498 | Guanosine 5'-Monophosphate | C06686 | C10H14N5O8P | Purine ribonucleoside monophosphates | 11340.00 | 12086.33 | 9167.00 | 11632.67 | 8419.33 | 10101.00 |
| 626 | Edaravone (Mci-186) | C07881 | C10H10N2O | Pyrazolones | 42692.00 | 26778.00 | 25022.00 | 37518.33 | 12077.67 | 62714.00 |
| 723 | 4-Pyridoxate | C00847 | C8H9NO4 | Pyridinecarboxylic acids | 10361.33 | 32115.67 | 15265.67 | 23359.00 | 61162.00 | 156917.00 |
| 2948 | Cdp | C00112 | C9H15N3O11P2 | Pyrimidine ribonucleoside diphosphates | 1426.33 | 29598.00 | 688.00 | 2367.67 | 10524.33 | 4771.00 |
| 2951 | Cytidine 5'-Diphosphate | C00112 | C9H15N3O11P2 | Pyrimidine ribonucleoside diphosphates | 7603.67 | 75729.33 | 6917.33 | 11736.00 | 24087.67 | 27402.00 |
| 2952 | Cytidine-5'-Diphosphate | C00112 | C9H15N3O11P2 | Pyrimidine ribonucleoside diphosphates | 22008.67 | 130219.00 | 24067.33 | 41268.00 | 71066.67 | 99487.67 |
| 139 | Uracil | C00106 | C4H4N2O2 | Pyrimidones | 14214.00 | 8472.00 | 8420.33 | 11186.00 | 10318.67 | 15811.33 |
| 136 | Cytosine | C00380 | C4H5N3O | Pyrimidones | 4138.00 | 3402.00 | 2554.33 | 3299.00 | 2724.33 | 3583.67 |
| 1012 | Xanthurenic Acid | C02470 | C10H7NO4 | Quinoline carboxylic acids | 1225.67 | 1592.33 | 2706.33 | 2639.67 | 12635.33 | 20976.67 |
| 2928 | Moxifloxacin | C07663 | C21H24FN3O4 | Quinoline carboxylic acids | 12603.33 | 9383.33 | 11257.00 | 21839.67 | 9112.33 | 342.33 |
| 5442 | S4 | C07593 | C29H48O15 | Saccharolipids | 1056.33 | 1441.00 | 2884.00 | 2492.00 | 1557.00 | 2244.67 |
| 360 | Salicyclic Acid | C00019 | C7H6O3 | Salicylic acids | 42477.67 | 66473.33 | 65841.33 | 77614.00 | 137522.33 | 97849.67 |
| 2879 | Ginkgolic Acid C17-1 | C13297 | C24H38O3 | Salicylic acids | 2890.33 | 2616.33 | 2398.00 | 3179.67 | 2122.00 | 3963.00 |
| 3491 | Scalarin | C06162 | C27H40O5 | Scalarane sesterterpenoids | 133731.67 | 312479.00 | 82396.00 | 244650.67 | 168593.67 | 245427.33 |
| 3245 | Terpestacin | C07127 | C25H38O4 | Secondary alcohols | 17230.00 | 16985.67 | 6389.00 | 13286.67 | 4291.00 | 6699.33 |
| 991 | Pantothenic Acid | C12661 | C9H17NO5 | Secondary alcohols | 5685.67 | 6529.00 | 2454.67 | 5743.33 | 3092.33 | 3863.33 |
| 4508 | Gossypol | C02283 | C30H30O8 | Sesquiterpenoids | 4846.67 | 2568.33 | 1832.00 | 4651.67 | 2036.00 | 8767.00 |
| 1060 | Humulene (Alpha) | C06199 | C15H24 | Sesquiterpenoids | 283514.33 | 4526580.33 | 151171.00 | 2707484.00 | 1850238.33 | 3844918.00 |
| 1881 | Shogaol | C10882 | C17H24O3 | Shogaols | 1521.67 | 1463.00 | 1205.33 | 2033.67 | 3196.67 | 5485.33 |
| 4766 | Sodium (E)-2-((4S)-16-Acetoxy-3,11-Dihydroxy-4,8,10,14-Tetramethyldodecahydro-1H-Cyclopenta[a]Phenanthren-17(2H,10H,14H)-Ylidene)-6-Methylhept-5-Enoate | C00093 | C31H47NaO6 | Steroid esters | 14789.00 | 15448.00 | 6632.00 | 13959.33 | 8284.67 | 17042.33 |
| 4912 | Furostane Base -2H + O-Hex | C08596 | C33H54O9 | Steroidal saponins | 3404.67 | 12085.00 | 4448.33 | 4193.00 | 6485.00 | 3873.33 |
| 6563 | Protodioscin | C11155 | C51H84O22 | Steroidal saponins | 19421.00 | 40606.67 | 27775.00 | 51629.00 | 44931.33 | 45473.33 |
| 2883 | Beta-Sitosterol | C01753 | C29H50O | Stigmastanes and derivatives | 20965.00 | 25217.33 | 9990.00 | 22741.00 | 1017.00 | 28353.33 |
| 1260 | Muramic Acid | C06470 | C9H17NO7 | Sugar acids and derivatives | 39300.67 | 46719.00 | 23126.33 | 56073.00 | 47452.33 | 72002.33 |
| 76 | Glycerol | C00025 | C3H8O3 | Sugar alcohols | 2779.67 | 2580.67 | 1201.67 | 2655.00 | 2005.33 | 2658.67 |
| 868 | Mannitol | C00392 | C6H14O6 | Sugar alcohols | 3078.67 | 4128.00 | 2575.00 | 4404.33 | 6062.67 | 3976.00 |
| 2561 | Omeprazole (Prilosec) | C12140 | C17H19N3O3S | Sulfinylbenzimidazoles | 3707.67 | 2677.67 | 1601.00 | 2547.33 | 1710.00 | 1675.67 |
| 1845 | Thymol-Beta-D-Glucoside | C09908 | C16H24O6 | Terpene glycosides | 3293.00 | 2840.33 | 1685.33 | 3331.33 | 2332.00 | 3348.00 |
| 43 | N,N-Dimethylformamide | C03134 | C3H7NO | Tertiary carboxylic acid amides | 10260.33 | 10036.33 | 13968.00 | 11161.67 | 12817.67 | 11986.67 |
| 1372 | Thiamine | C07130 | C12H17N4OS | Thiamines | 45933.00 | 11358.67 | 12510.67 | 24344.33 | 28869.67 | 41276.67 |
| 3607 | Alpha-Tocopherol | C02477 | C29H50O2 | Tocopherols | 4065.67 | 5469.00 | 3473.00 | 5568.00 | 3045.00 | 2544.00 |
| 2947 | Delta-Tocopherol | C14151 | C27H46O2 | Tocopherols | 5083.33 | 4704.33 | 2405.67 | 5462.67 | 2642.67 | 3223.33 |
| 3247 | Alpha-Tocotrienol | C14153 | C29H44O2 | Tocotrienols | 5107.00 | 4841.00 | 3115.33 | 6796.67 | 3777.67 | 4304.67 |
| 4853 | Glyceryl Tridecanoate | C00064 | C33H62O6 | Triacylglycerols | 8061.33 | 9357.33 | 4503.67 | 7219.00 | 3910.33 | 6358.67 |
| 2898 | Tri(Butoxyethyl) Phosphate | C01083 | C18H39O7P | Trialkyl phosphates | 3583.33 | 5265.33 | 3212.00 | 4968.67 | 3117.33 | 6315.67 |
| 2971 | Diacetoxyscirpenol | C09662 | C19H26O7 | Trichothecenes | 16909.67 | 14817.67 | 9165.67 | 22291.33 | 5852.00 | 10513.00 |
| 2626 | Cholic Acid | C00695 | C24H40O5 | Trihydroxy bile acids, alcohols and derivatives | 23752.67 | 11869.67 | 5470.33 | 12904.67 | 14593.00 | 11209.00 |
| 3516 | Cholic Acid | C00695 | C24H40O5 | Trihydroxy bile acids, alcohols and derivatives | 2177.67 | 4037.33 | 2144.33 | 3289.00 | 3788.33 | 6061.67 |
| 6399 | Hosenkoside G | C00155 | C47H80O19 | Triterpenoids | 17987.67 | 33536.67 | 6187.00 | 10762.33 | 4205.67 | 5920.67 |
| 3772 | Pristimerin | C01953 | C30H40O4 | Triterpenoids | 4698.67 | 4231.67 | 2915.00 | 5387.33 | 2573.33 | 5714.67 |
| 4008 | Quillaic Acid | C08257 | C30H46O5 | Triterpenoids | 5134.67 | 7236.33 | 2531.00 | 5188.00 | 2884.00 | 4161.00 |
| 3272 | Beta-Amyrin | C08616 | C30H50O | Triterpenoids | 600.00 | 497.67 | 419.67 | 409.67 | 498.00 | 445.33 |
| 4546 | Ganoderenic Acid C | C10062 | C30H44O7 | Triterpenoids | 9161.67 | 10266.67 | 3748.33 | 10914.67 | 489.00 | 5932.67 |
| 3916 | Beta-Elemonic Acid | C16927 | C30H46O3 | Triterpenoids | 5862.33 | 39588.00 | 4806.33 | 36626.00 | 31499.33 | 47789.00 |
| 4695 | Ganoderic Acid D2 | C19654 | C30H42O8 | Triterpenoids | 141420.67 | 241096.33 | 126948.67 | 192620.67 | 55864.67 | 48431.00 |
| 3406 | Colchicine | C07592 | C22H25NO6 | Tropones | 11335.33 | 7914.33 | 4074.67 | 8438.33 | 11536.67 | 17713.33 |
| 844 | 3,4-Dihydroxy-L-Phenylalanine | C00355 | C9H11NO4 | Tyrosine and derivatives | 2166.67 | 1993.67 | 1447.67 | 2698.00 | 1236.00 | 1547.33 |
| 41 | Urea | C00086 | CH4N2O | Ureas | 26360.67 | 31778.67 | 43628.67 | 36120.00 | 31499.00 | 43304.00 |
| 3562 | Vitamin K1 | C05850 | C31H46O2 | Vitamin K compounds | 30066.00 | 22396.33 | 19891.67 | 35736.00 | 9883.00 | 11330.00 |
| 697 | Theophylline | C01047 | C7H8N4O2 | Xanthines | 2717.33 | 6424.33 | 2559.67 | 5852.33 | 1672.33 | 5293.00 |
| 969 | Caffeine | C07481 | C8H10N4O2 | Xanthines | 7461.33 | 5814.33 | 11273.33 | 14329.00 | 12278.67 | 9035.67 |
| 589 | 1-Methylxanthine | C16358 | C6H6N4O2 | Xanthines | 26046.33 | 19750.00 | 5380.00 | 14450.33 | 13672.67 | 10292.33 |
| 6286 | Oscillaxanthin | C00295 | C52H76O12 | Xanthophylls | 60655.00 | 104708.00 | 15599.00 | 24162.33 | 9449.67 | 16331.67 |
| 5470 | Fucoxanthin | C00858 | C42H58O6 | Xanthophylls | 8684.33 | 8375.00 | 4614.67 | 9189.67 | 5245.00 | 6291.67 |
| 4947 | Antheraxanthin | C08579 | C40H56O3 | Xanthophylls | 188461.00 | 207052.33 | 87601.33 | 158281.33 | 84912.33 | 125747.67 |
| 5056 | Astaxanthin | C08580 | C40H52O4 | Xanthophylls | 23004.33 | 17230.00 | 11222.00 | 20636.67 | 10262.33 | 9539.33 |
| 4787 | Canthaxanthin | C08583 | C40H52O2 | Xanthophylls | 56760.00 | 67882.67 | 27690.67 | 54669.33 | 24800.00 | 44396.00 |

| TableS5. Length distribution of highly enriched siRNAs. | | | | | | | | |
| --- | --- | --- | --- | --- | --- | --- | --- | --- |
| Number | 21nt | ratio(%) | 22nt | ratio(%) | 23nt | ratio(%) | 24nt | ratio(%) |
| DTS_108 | 535 | 70.03 | 157 | 20.55 | 64 | 8.38 | 8 | 1.05 |
| DTS_123 | 533 | 71.16 | 152 | 20.29 | 56 | 7.48 | 8 | 1.07 |
| DTS_127 | 522 | 77.45 | 114 | 16.91 | 30 | 4.45 | 8 | 1.19 |
| TJ806 | 58 | 74.36 | 20 | 25.64 | 0 | 0.00 | 0 | 0.00 |
| AR02 | 42 | 100.00 | 0 | 0.00 | 0 | 0.00 | 0 | 0.00 |
| AR03 | 62 | 100.00 | 0 | 0.00 | 0 | 0.00 | 0 | 0.00 |

| Table S6. The abundance of siRNAs enriched in maize transcripts and their potential off-target genes. | | | | | | | | | | |
| --- | --- | --- | --- | --- | --- | --- | --- | --- | --- | --- |
| maize-transcription | mis-match | dsRNA_position | sequence | DTS_108 | DTS_123 | DTS_127 | AR02 | AR03 | TJ806 | jellyfish |
| Zm00001d014451_T001 | 2 | 31 | AGCCGCTGAGGAACTGAAGAA | 284.3 | 228.3 | 274.7 | - | - | 2.0 | yes |
| Zm00001d014451_T002 | 2 | 31 | AGCCGCTGAGGAACTGAAGAA | 284.3 | 228.3 | 274.7 | - | - | 2.0 | yes |
| Zm00001d014451_T001 | 2 | 31 | AGCCGCTGAGGAACTGAAGAAG | 5.0 | 5.3 | 4.5 | - | - | - | yes |
| Zm00001d014451_T002 | 2 | 31 | AGCCGCTGAGGAACTGAAGAAG | 5.0 | 5.3 | 4.5 | - | - | - | yes |
| Zm00001d014451_T001 | 2 | 32 | GCCGCTGAGGAACTGAAGAAG | 182.0 | 114.7 | 207.0 | - | - | - | yes |
| Zm00001d014451_T002 | 2 | 32 | GCCGCTGAGGAACTGAAGAAG | 182.0 | 114.7 | 207.0 | - | - | - | yes |
| Zm00001d026704_T001 | 2 | 32 | GCCGCTGAGGAACTGAAGAAG | 182.0 | 114.7 | 207.0 | - | - | - | yes |
| Zm00001d026704_T002 | 2 | 32 | GCCGCTGAGGAACTGAAGAAG | 182.0 | 114.7 | 207.0 | - | - | - | yes |
| Zm00001d031128_T001 | 2 | 33 | CCGCTGAGGAACTGAAGAAGG | 2732.0 | 2203.3 | 4604.0 | 1.5 | 5.0 | 8.0 | yes |
| Zm00001d031128_T002 | 2 | 33 | CCGCTGAGGAACTGAAGAAGG | 2732.0 | 2203.3 | 4604.0 | 1.5 | 5.0 | 8.0 | yes |
| Zm00001d031128_T003 | 2 | 33 | CCGCTGAGGAACTGAAGAAGG | 2732.0 | 2203.3 | 4604.0 | 1.5 | 5.0 | 8.0 | yes |
| Zm00001d031128_T004 | 2 | 33 | CCGCTGAGGAACTGAAGAAGG | 2732.0 | 2203.3 | 4604.0 | 1.5 | 5.0 | 8.0 | yes |
| Zm00001d042564_T001 | 2 | 33 | CCGCTGAGGAACTGAAGAAGG | 2732.0 | 2203.3 | 4604.0 | 1.5 | 5.0 | 8.0 | yes |
| Zm00001d042568_T001 | 2 | 33 | CCGCTGAGGAACTGAAGAAGG | 2732.0 | 2203.3 | 4583.3 | 1963.0 | 1963.0 | 1963.0 | yes |
| Zm00001d031933_T001 | 2 | 34 | CGCTGAGGAACTGAAGAAGGA | 320.7 | 170.7 | 279.0 | - | 1.0 | 2.0 | yes |
| Zm00001d031933_T006 | 2 | 34 | CGCTGAGGAACTGAAGAAGGA | 320.7 | 170.7 | 279.0 | - | 1.0 | 2.0 | yes |
| Zm00001d031933_T007 | 2 | 34 | CGCTGAGGAACTGAAGAAGGA | 320.7 | 170.7 | 279.0 | - | 1.0 | 2.0 | yes |
| Zm00001d031933_T008 | 2 | 34 | CGCTGAGGAACTGAAGAAGGA | 320.7 | 170.7 | 279.0 | - | 1.0 | 2.0 | yes |
| Zm00001d031933_T009 | 2 | 34 | CGCTGAGGAACTGAAGAAGGA | 320.7 | 170.7 | 279.0 | - | 1.0 | 2.0 | yes |
| Zm00001d031933_T001 | 2 | 35 | GCTGAGGAACTGAAGAAGGAG | 205.0 | 152.0 | 175.0 | - | - | - | yes |
| Zm00001d031933_T006 | 2 | 35 | GCTGAGGAACTGAAGAAGGAG | 205.0 | 152.0 | 175.0 | - | - | - | yes |
| Zm00001d031933_T007 | 2 | 35 | GCTGAGGAACTGAAGAAGGAG | 205.0 | 152.0 | 175.0 | - | - | - | yes |
| Zm00001d031933_T008 | 2 | 35 | GCTGAGGAACTGAAGAAGGAG | 205.0 | 152.0 | 175.0 | - | - | - | yes |
| Zm00001d031933_T009 | 2 | 35 | GCTGAGGAACTGAAGAAGGAG | 205.0 | 152.0 | 175.0 | - | - | - | yes |
| Zm00001d042564_T001 | 2 | 35 | GCTGAGGAACTGAAGAAGGAG | 205.0 | 152.0 | 175.0 | - | - | - | yes |
| Zm00001d042568_T001 | 2 | 35 | GCTGAGGAACTGAAGAAGGAG | 205.0 | 152.0 | 175.0 | - | - | - | yes |
| Zm00001d006628_T005 | 2 | 38 | GAGGAACTGAAGAAGGAGCAG | 310.3 | 201.0 | 212.7 | - | - | 1.0 | yes |
| Zm00001d006881_T002 | 2 | 38 | GAGGAACTGAAGAAGGAGCAG | 310.3 | 201.0 | 212.7 | - | - | 1.0 | yes |
| Zm00001d006881_T002 | 2 | 38 | GAGGAACTGAAGAAGGAGCAG | 310.3 | 201.0 | 212.7 | - | - | 1.0 | yes |
| Zm00001d006881_T003 | 2 | 38 | GAGGAACTGAAGAAGGAGCAG | 310.3 | 201.0 | 212.7 | - | - | 1.0 | yes |
| Zm00001d006881_T003 | 2 | 38 | GAGGAACTGAAGAAGGAGCAG | 310.3 | 201.0 | 212.7 | - | - | 1.0 | yes |
| Zm00001d006881_T005 | 2 | 38 | GAGGAACTGAAGAAGGAGCAG | 310.3 | 201.0 | 212.7 | - | - | 1.0 | yes |
| Zm00001d006881_T005 | 2 | 38 | GAGGAACTGAAGAAGGAGCAG | 310.3 | 201.0 | 212.7 | - | - | 1.0 | yes |
| Zm00001d006881_T007 | 2 | 38 | GAGGAACTGAAGAAGGAGCAG | 310.3 | 201.0 | 212.7 | - | - | 1.0 | yes |
| Zm00001d006881_T007 | 2 | 38 | GAGGAACTGAAGAAGGAGCAG | 310.3 | 201.0 | 212.7 | - | - | 1.0 | yes |
| Zm00001d006881_T009 | 2 | 38 | GAGGAACTGAAGAAGGAGCAG | 310.3 | 201.0 | 212.7 | - | - | 1.0 | yes |
| Zm00001d006881_T009 | 2 | 38 | GAGGAACTGAAGAAGGAGCAG | 310.3 | 201.0 | 212.7 | - | - | 1.0 | yes |
| Zm00001d006881_T011 | 2 | 38 | GAGGAACTGAAGAAGGAGCAG | 310.3 | 201.0 | 212.7 | - | - | 1.0 | yes |
| Zm00001d006881_T011 | 2 | 38 | GAGGAACTGAAGAAGGAGCAG | 310.3 | 201.0 | 212.7 | - | - | 1.0 | yes |
| Zm00001d006881_T013 | 2 | 38 | GAGGAACTGAAGAAGGAGCAG | 310.3 | 201.0 | 212.7 | - | - | 1.0 | yes |
| Zm00001d006881_T013 | 2 | 38 | GAGGAACTGAAGAAGGAGCAG | 310.3 | 201.0 | 212.7 | - | - | 1.0 | yes |
| Zm00001d007394_T001 | 2 | 38 | GAGGAACTGAAGAAGGAGCAG | 310.3 | 201.0 | 212.7 | - | - | 1.0 | yes |
| Zm00001d014294_T023 | 2 | 38 | GAGGAACTGAAGAAGGAGCAG | 310.3 | 201.0 | 212.7 | - | - | 1.0 | yes |
| Zm00001d041443_T004 | 2 | 38 | GAGGAACTGAAGAAGGAGCAG | 310.3 | 201.0 | 212.7 | - | - | 1.0 | yes |
| Zm00001d041443_T009 | 2 | 38 | GAGGAACTGAAGAAGGAGCAG | 310.3 | 201.0 | 212.7 | - | - | 1.0 | yes |
| Zm00001d041443_T012 | 2 | 38 | GAGGAACTGAAGAAGGAGCAG | 310.3 | 201.0 | 212.7 | - | - | 1.0 | yes |
| Zm00001d041443_T013 | 2 | 38 | GAGGAACTGAAGAAGGAGCAG | 310.3 | 201.0 | 212.7 | - | - | 1.0 | yes |
| Zm00001d041443_T021 | 2 | 38 | GAGGAACTGAAGAAGGAGCAG | 310.3 | 201.0 | 212.7 | - | - | 1.0 | yes |
| Zm00001d041443_T022 | 2 | 38 | GAGGAACTGAAGAAGGAGCAG | 310.3 | 201.0 | 212.7 | - | - | 1.0 | yes |
| Zm00001d041443_T023 | 2 | 38 | GAGGAACTGAAGAAGGAGCAG | 310.3 | 201.0 | 212.7 | - | - | 1.0 | yes |

| TableS7. Enrichment of the first base of siRNA in the Top 100 bases of dsRNA | | | | | | |
| --- | --- | --- | --- | --- | --- | --- |
| position | DTS_108 | DTS_123 | DTS_127 | TJ806 | AR02 | AR03 |
| 1 | 0 | 0 | 0 | 0 | 0 | 0 |
| 2 | 0 | 0 | 0 | 0 | 0 | 0 |
| 3 | 0 | 0 | 0 | 0 | 0 | 0 |
| 4 | 0 | 0 | 0 | 0 | 0 | 0 |
| 5 | 0 | 0 | 0 | 0 | 0 | 0 |
| 6 | 0 | 0 | 0 | 0 | 0 | 0 |
| 7 | 0 | 0 | 0 | 0 | 0 | 0 |
| 8 | 0 | 0 | 0 | 0 | 0 | 0 |
| 9 | 0 | 0 | 0 | 0 | 0 | 0 |
| 10 | 0 | 0 | 0 | 0 | 0 | 0 |
| 11 | 0 | 0 | 0 | 0 | 0 | 0 |
| 12 | 0 | 0 | 0 | 0 | 0 | 0 |
| 13 | 0 | 0 | 0 | 0 | 0 | 0 |
| 14 | 0 | 0 | 0 | 0 | 0 | 0 |
| 15 | 0 | 0 | 0 | 0 | 0 | 0 |
| 16 | 0 | 0 | 0 | 0 | 0 | 0 |
| 17 | 0 | 0 | 0 | 0 | 0 | 0 |
| 18 | 0 | 0 | 0 | 0 | 0 | 0 |
| 19 | 0 | 0 | 0 | 0 | 0 | 0 |
| 20 | 0 | 0 | 0 | 0 | 0 | 0 |
| 21 | 0 | 0 | 0 | 0 | 0 | 0 |
| 22 | 0 | 0 | 0 | 0 | 0 | 0 |
| 23 | 0 | 0 | 0 | 0 | 0 | 0 |
| 24 | 0 | 0 | 0 | 0 | 0 | 0 |
| 25 | 0 | 0 | 0 | 0 | 0 | 0 |
| 26 | 0 | 0 | 0 | 0 | 0 | 0 |
| 27 | 0 | 0 | 0 | 0 | 0 | 0 |
| 28 | 0 | 0 | 0 | 0 | 0 | 0 |
| 29 | 0 | 0 | 0 | 0 | 0 | 0 |
| 30 | 0 | 0 | 0 | 0 | 0 | 0 |
| 31 | 579 | 467 | 555 | 1 | 0 | 0 |
| 32 | 728 | 459 | 828 | 0 | 0 | 0 |
| 33 | 16392 | 13220 | 27624 | 32 | 6 | 20 |
| 34 | 1603 | 853 | 1395 | 7 | 0 | 0 |
| 35 | 1435 | 1064 | 1225 | 0 | 0 | 0 |
| 36 | 0 | 0 | 0 | 0 | 0 | 0 |
| 37 | 0 | 0 | 0 | 0 | 0 | 0 |
| 38 | 7852 | 5096 | 5389 | 8 | 0 | 0 |
| 39 | 7991 | 5885 | 8237 | 6 | 0 | 0 |
| 40 | 411 | 126 | 426 | 0 | 0 | 0 |
| 41 |  | 63 | 146 | 0 | 0 | 0 |
| 42 | 1224 | 833 | 1558 | 0 | 0 | 0 |
| 43 | 0 | 0 | 0 | 0 | 0 | 0 |
| 44 | 0 | 0 | 0 | 0 | 0 | 0 |
| 45 | 0 | 0 | 0 | 0 | 0 | 0 |
| 46 | 175 | 97 | 107 | 0 | 0 | 0 |
| 47 | 1146 | 528 | 842 | 0 | 0 | 0 |
| 48 | 4102 | 2429 | 3446 | 9 | 0 | 5 |
| 49 | 156 | 117 | 148 | 0 | 0 | 0 |
| 50 | 0 | 0 | 0 | 0 | 0 | 0 |
| 51 | 0 | 0 | 0 | 0 | 0 | 0 |
| 52 | 0 | 0 | 0 | 0 | 0 | 0 |
| 53 | 0 | 0 | 0 | 0 | 0 | 0 |
| 54 | 0 | 0 | 0 | 0 | 0 | 0 |
| 55 | 0 | 0 | 0 | 0 | 0 | 0 |
| 56 | 0 | 0 | 0 | 0 | 0 | 0 |
| 57 | 0 | 0 | 0 | 0 | 0 | 0 |
| 58 | 0 | 0 | 0 | 0 | 0 | 0 |
| 59 | 0 | 0 | 0 | 0 | 0 | 0 |
| 60 | 0 | 0 | 0 | 0 | 0 | 0 |
| 61 | 619 | 192 | 506 | 0 | 0 | 0 |
| 62 | 71 | 53 | 47 | 0 | 0 | 0 |
| 63 | 0 | 0 | 0 | 0 | 0 | 0 |
| 64 | 0 | 0 | 0 | 0 | 0 | 0 |
| 65 | 0 | 0 | 0 | 0 | 0 | 0 |
| 66 | 0 | 0 | 0 | 0 | 0 | 0 |
| 67 | 74 | 42 | 54 | 0 | 0 | 0 |
| 68 | 0 | 0 | 0 | 0 | 0 | 0 |
| 69 | 0 | 0 | 0 | 0 | 0 | 0 |
| 70 | 0 | 0 | 0 | 0 | 0 | 0 |
| 71 | 0 | 0 | 0 | 0 | 0 | 0 |
| 72 | 0 | 0 | 0 | 0 | 0 | 0 |
| 73 | 0 | 0 | 0 | 0 | 0 | 0 |
| 74 | 0 | 0 | 0 | 0 | 0 | 0 |
| 75 | 0 | 0 | 0 | 0 | 0 | 0 |
| 76 | 1182 | 639 | 726 | 3 | 1 |  |
| 77 | 0 | 0 | 0 | 0 | 0 | 0 |
| 78 | 0 | 0 | 0 | 0 | 0 | 0 |
| 79 | 0 | 0 | 0 | 0 | 0 | 0 |
| 80 | 0 | 0 | 0 | 0 | 0 | 0 |
| 81 | 0 | 0 | 0 | 0 | 0 | 0 |
| 82 | 0 | 0 | 0 | 0 | 0 | 0 |
| 83 | 0 | 0 | 0 | 0 | 0 | 0 |
| 84 | 0 | 0 | 0 | 0 | 0 | 0 |
| 85 | 0 | 0 | 0 | 0 | 0 | 0 |
| 86 | 12885 | 5796 | 7563 | 9 | 0 | 18 |
| 87 | 7359 | 4593 | 4542 | 0 | 15 | 12 |
| 88 | 516 | 174 | 423 | 3 | 0 |  |
| 89 | 0 | 0 | 0 | 0 | 0 | 0 |
| 90 | 0 | 0 | 0 | 0 | 0 | 0 |
| 91 | 0 | 0 | 0 | 0 | 0 | 0 |
| 92 | 0 | 0 | 0 | 0 | 0 | 0 |
| 93 | 0 | 0 | 0 | 0 | 0 | 0 |
| 94 | 0 | 0 | 0 | 0 | 0 | 0 |
| 95 | 0 | 0 | 0 | 0 | 0 | 0 |
| 96 | 0 | 0 | 0 | 0 | 0 | 0 |
| 97 | 0 | 0 | 0 | 0 | 0 | 0 |
| 98 | 0 | 0 | 0 | 0 | 0 | 0 |
| 99 | 0 | 0 | 0 | 0 | 0 | 0 |
| 100 | 0 | 0 | 0 | 0 | 0 | 0 |

| Table S8. Partly of the expression of detected genes in different corn lines. | | | | | | |
| --- | --- | --- | --- | --- | --- | --- |
| Gene ID | FPKM value | | | | | |
|  | DTS_108 | DTS_123 | DTS_127 | TJ806 | AR02 | AR03 |
| GRMZM5G800096 | 0.135 | 0.061 | 0.123 | 0.070 | 0.114 | 0.097 |
| GRMZM5G800101 | 0.000 | 0.000 | 0.000 | 0.002 | 0.001 | 0.001 |
| GRMZM5G800457 | 0.000 | 0.000 | 0.000 | 0.016 | 0.000 | 0.000 |
| GRMZM5G800980 | 0.079 | 0.059 | 0.085 | 0.062 | 0.045 | 0.078 |
| GRMZM5G801958 | 0.020 | 0.020 | 0.014 | 0.009 | 0.000 | 0.006 |
| GRMZM5G804358 | 0.680 | 0.175 | 0.177 | 0.161 | 0.165 | 0.077 |
| GRMZM5G804708 | 0.078 | 0.062 | 0.069 | 0.050 | 0.034 | 0.038 |
| GRMZM5G804776 | 0.000 | 0.113 | 0.000 | 0.000 | 0.000 | 0.000 |
| GRMZM5G805421 | 0.000 | 0.000 | 0.000 | 0.071 | 0.000 | 0.069 |
| GRMZM5G806435 | 0.003 | 0.000 | 0.000 | 0.000 | 0.002 | 0.007 |
| GRMZM5G806488 | 0.014 | 0.011 | 0.015 | 0.022 | 0.014 | 0.037 |
| GRMZM5G807592 | 0.212 | 0.000 | 0.359 | 0.130 | 0.202 | 0.248 |
| GRMZM5G808402 | 0.020 | 0.018 | 0.006 | 0.006 | 0.011 | 0.003 |
| GRMZM5G809869 | 0.017 | 0.019 | 0.000 | 0.033 | 0.018 | 0.000 |
| GRMZM5G810298 | 0.000 | 0.010 | 0.031 | 0.015 | 0.009 | 0.013 |
| GRMZM5G811749 | 0.315 | 0.244 | 0.338 | 0.259 | 0.230 | 0.181 |
| GRMZM5G813608 | 0.003 | 0.003 | 0.000 | 0.002 | 0.003 | 0.005 |
| GRMZM5G814594 | 0.272 | 0.000 | 0.000 | 0.000 | 0.000 | 0.000 |
| GRMZM5G815453 | 0.700 | 0.847 | 0.619 | 0.607 | 0.975 | 0.818 |
| GRMZM5G815553 | 1.658 | 0.000 | 0.000 | 0.000 | 0.000 | 0.000 |
| GRMZM5G815606 | 0.162 | 0.000 | 0.208 | 0.120 | 0.052 | 0.030 |
| GRMZM5G816453 | 0.000 | 0.000 | 0.015 | 0.000 | 0.000 | 0.013 |
| GRMZM5G816772 | 0.000 | 0.000 | 0.000 | 0.000 | 0.000 | 0.008 |
| GRMZM5G818111 | 0.000 | 0.000 | 0.031 | 0.000 | 0.000 | 0.000 |
| GRMZM5G819109 | 0.000 | 0.373 | 0.000 | 0.000 | 0.000 | 0.000 |
| GRMZM5G821527 | 0.000 | 0.060 | 0.000 | 0.000 | 0.015 | 0.000 |
| GRMZM5G821687 | 0.077 | 0.000 | 0.000 | 0.073 | 0.074 | 0.097 |
| GRMZM5G825253 | 0.000 | 0.805 | 0.320 | 0.429 | 0.000 | 0.388 |
| GRMZM5G831399 | 0.148 | 0.000 | 0.000 | 0.034 | 0.027 | 0.000 |
| GRMZM5G834128 | 0.000 | 0.000 | 0.017 | 0.000 | 0.000 | 0.007 |
| GRMZM5G834666 | 0.346 | 0.096 | 0.000 | 0.488 | 0.305 | 0.000 |
| GRMZM5G838963 | 0.000 | 0.000 | 0.000 | 0.000 | 0.000 | 0.017 |
| GRMZM5G839924 | 0.036 | 0.000 | 0.000 | 0.000 | 0.000 | 0.000 |
| GRMZM5G844030 | 0.000 | 0.000 | 0.000 | 0.000 | 0.014 | 0.013 |
| GRMZM5G845244 | 0.205 | 0.067 | 0.050 | 0.067 | 0.186 | 0.070 |
| GRMZM5G848448 | 0.267 | 0.000 | 0.000 | 0.000 | 0.000 | 0.000 |
| GRMZM5G851130 | 0.023 | 0.000 | 0.008 | 0.000 | 0.000 | 0.000 |
| GRMZM5G851769 | 0.246 | 0.032 | 0.046 | 0.030 | 0.033 | 0.116 |
| GRMZM5G853305 | 0.135 | 0.000 | 0.000 | 0.129 | 0.128 | 0.000 |
| GRMZM5G853723 | 0.000 | 0.000 | 0.000 | 0.006 | 0.000 | 0.012 |
| GRMZM5G854065 | 0.000 | 0.000 | 0.068 | 0.000 | 0.025 | 0.000 |
| GRMZM5G856777 | 0.220 | 0.179 | 0.214 | 0.114 | 0.116 | 0.096 |
| GRMZM5G858556 | 0.077 | 0.042 | 0.060 | 0.068 | 0.069 | 0.019 |
| GRMZM5G861212 | 0.000 | 0.000 | 0.285 | 0.000 | 0.289 | 0.000 |
| GRMZM5G861791 | 0.025 | 0.000 | 0.011 | 0.003 | 0.010 | 0.003 |
| GRMZM5G862955 | 0.268 | 0.205 | 0.330 | 0.286 | 0.209 | 0.138 |
| GRMZM5G864407 | 0.088 | 0.043 | 0.094 | 0.085 | 0.000 | 0.000 |
| GRMZM5G866223 | 0.000 | 0.000 | 0.000 | 0.041 | 0.111 | 0.000 |
| GRMZM5G867512 | 0.291 | 0.046 | 0.082 | 0.113 | 0.059 | 0.060 |
| GRMZM5G874448 | 0.086 | 0.116 | 0.091 | 0.023 | 0.039 | 0.079 |
| GRMZM5G875287 | 0.061 | 0.039 | 0.058 | 0.035 | 0.027 | 0.037 |
| GRMZM5G876106 | 0.000 | 0.006 | 0.000 | 0.000 | 0.000 | 0.000 |
| GRMZM5G877040 | 0.000 | 0.067 | 0.168 | 0.000 | 0.000 | 0.000 |
| GRMZM5G881135 | 0.382 | 0.000 | 0.000 | 0.482 | 0.306 | 0.117 |
| GRMZM5G884538 | 0.377 | 0.175 | 0.274 | 0.147 | 0.303 | 0.103 |
| GRMZM5G884707 | 0.012 | 0.000 | 0.000 | 0.008 | 0.011 | 0.000 |
| GRMZM5G884912 | 0.327 | 0.238 | 0.244 | 0.422 | 0.116 | 0.416 |
| GRMZM5G889036 | 0.178 | 0.084 | 0.111 | 0.044 | 0.213 | 0.039 |
| GRMZM5G889299 | 0.004 | 0.008 | 0.004 | 0.000 | 0.020 | 0.027 |
| GRMZM5G889790 | 0.386 | 0.000 | 0.000 | 0.195 | 0.166 | 0.000 |
| GRMZM5G889905 | 0.113 | 0.000 | 0.026 | 0.000 | 0.000 | 0.035 |
| GRMZM5G890451 | 0.246 | 0.181 | 0.130 | 0.057 | 0.000 | 0.154 |
| GRMZM5G892247 | 0.000 | 0.002 | 0.000 | 0.000 | 0.000 | 0.004 |
| GRMZM5G892769 | 0.000 | 0.000 | 0.004 | 0.011 | 0.000 | 0.008 |
| GRMZM5G894515 | 0.006 | 0.012 | 0.009 | 0.005 | 0.007 | 0.004 |
| GRMZM5G898647 | 0.000 | 0.054 | 0.000 | 0.000 | 0.046 | 0.000 |
| GRMZM5G899892 | 0.000 | 0.000 | 0.000 | 0.000 | 0.204 | 0.000 |
| Zm00001d001763 | 2.851 | 1.694 | 2.268 | 2.260 | 1.600 | 2.187 |
| Zm00001d001765 | 5.377 | 4.629 | 4.828 | 3.852 | 4.573 | 7.432 |
| Zm00001d001766 | 100.390 | 146.799 | 113.510 | 103.888 | 85.741 | 85.848 |
| Zm00001d001767 | 1.156 | 0.871 | 1.133 | 1.407 | 1.222 | 0.291 |
| Zm00001d001769 | 0.771 | 1.152 | 1.189 | 1.707 | 0.231 | 0.238 |
| Zm00001d001770 | 0.200 | 0.227 | 0.229 | 0.259 | 3.729 | 0.254 |
| Zm00001d001771 | 21.481 | 16.748 | 18.583 | 26.218 | 0.253 | 25.133 |
| Zm00001d001772 | 0.449 | 0.489 | 0.255 | 0.563 | 0.326 | 0.504 |
| Zm00001d001773 | 0.071 | 0.082 | 0.103 | 0.083 | 0.008 | 0.122 |
| Zm00001d001774 | 44.341 | 46.701 | 43.653 | 30.865 | 6.466 | 33.015 |
| Zm00001d001779 | 5.246 | 5.083 | 4.742 | 6.039 | 2.997 | 10.696 |
| Zm00001d001780 | 4.601 | 3.189 | 4.112 | 2.987 | 5.043 | 4.541 |
| Zm00001d001781 | 9.659 | 7.409 | 10.806 | 7.449 | 6.651 | 15.228 |
| Zm00001d001784 | 18.354 | 15.108 | 18.277 | 12.838 | 8.534 | 23.060 |
| Zm00001d001785 | 0.125 | 0.246 | 0.125 | 0.122 | 0.083 | 0.104 |
| Zm00001d001786 | 0.744 | 0.708 | 0.711 | 0.813 | 0.785 | 0.371 |
| Zm00001d001787 | 7.394 | 6.250 | 6.690 | 6.571 | 4.883 | 6.978 |
| Zm00001d001788 | 11.855 | 11.318 | 8.855 | 8.795 | 12.805 | 15.075 |
| Zm00001d001789 | 3.463 | 3.095 | 3.063 | 2.918 | 6.438 | 3.808 |
| Zm00001d001790 | 6.243 | 5.096 | 4.880 | 4.736 | 4.414 | 4.713 |
| Zm00001d001791 | 0.000 | 0.000 | 0.000 | 0.044 | 0.077 | 0.134 |
| Zm00001d001792 | 0.020 | 0.009 | 0.000 | 0.019 | 0.010 | 0.074 |
| Zm00001d001796 | 0.013 | 0.031 | 0.000 | 0.000 | 0.000 | 0.000 |
| Zm00001d001797 | 2.205 | 1.443 | 1.853 | 1.625 | 0.499 | 1.546 |
| Zm00001d001798 | 14.595 | 11.878 | 13.167 | 12.679 | 13.710 | 12.941 |
| Zm00001d001799 | 1.201 | 0.838 | 1.222 | 1.022 | 0.697 | 1.259 |
| Zm00001d001800 | 2.479 | 2.359 | 2.063 | 2.312 | 0.918 | 1.933 |
| Zm00001d001802 | 4.718 | 6.674 | 5.518 | 5.952 | 2.227 | 1.642 |
| Zm00001d001803 | 0.363 | 0.273 | 0.381 | 0.297 | 0.330 | 0.388 |
| Zm00001d001804 | 0.228 | 0.144 | 0.273 | 0.229 | 0.454 | 0.224 |
| Zm00001d001806 | 14.237 | 10.286 | 11.179 | 9.463 | 11.250 | 12.680 |
| Zm00001d001807 | 5.397 | 5.090 | 5.670 | 5.682 | 4.436 | 4.436 |
| Zm00001d001808 | 4.758 | 3.631 | 4.258 | 4.298 | 3.532 | 4.229 |

| Table S9. Number of DEGs and percentage of the DEGs of the total detected genes in pairwise comparisons of different maize lines. | | | | | | | | |
| --- | --- | --- | --- | --- | --- | --- | --- | --- |
| Group | Comparisons | Total DEGs | UP | up raito | DOWN | down ratio | Unchanged genes | Percentage of DEGs on the total detected genes (%) |
| Group1 | DTS-108/TJ806 | 70 | 27 | 38.57% | 43 | 61.43% | 51957 | 0.13% |
|  | DTS-123/TJ806 | 50 | 27 | 54.00% | 23 | 46.00% | 52003 | 0.10% |
|  | DTS-127/TJ806 | 77 | 25 | 32.47% | 52 | 67.53% | 52013 | 0.15% |
| Group2 | AR02/AR03 | 1292 | 585 | 45.28% | 707 | 54.72% | 51117 | 2.53% |
|  | AR02/TJ806 | 4344 | 1870 | 43.05% | 2474 | 56.95% | 51520 | 8.43% |
|  | AR03/TJ806 | 4497 | 1997 | 44.41% | 2500 | 55.59% | 51401 | 8.75% |
| Group3 | DTS-108/DTS-123 | 119 | 17 | 14.29% | 102 | 85.71% | 52156 | 0.23% |
|  | DTS-108/DTS-127 | 8 | 4 | 50.00% | 4 | 50.00% | 52166 | 0.02% |
|  | DTS-123/DTS-127 | 72 | 57 | 79.17% | 15 | 20.83% | 52212 | 0.14% |
| Group4 | DTS-108/AR02 | 4628 | 2446 | 52.85% | 2182 | 47.15% | 51673 | 8.96% |
|  | DTS-123/AR02 | 4419 | 2526 | 57.16% | 1893 | 42.84% | 51719 | 8.54% |
|  | DTS-127/AR02 | 4506 | 2457 | 54.53% | 2049 | 45.47% | 51729 | 8.71% |
|  | DTS-108/AR03 | 4687 | 2363 | 50.42% | 2324 | 49.58% | 51554 | 9.09% |
|  | DTS-123/AR03 | 4387 | 2463 | 56.14% | 1924 | 43.86% | 51600 | 8.50% |
|  | DTS-127/AR03 | 4765 | 2517 | 52.82% | 2248 | 47.18% | 51610 | 9.23% |

| Table S10. Top 10 terms of Gene Ontology enrichment analyses of DEGs among different comparisons. | | | | | | | |
| --- | --- | --- | --- | --- | --- | --- | --- |
| Group | Pairwise | GO ID | Descrption | Num | Qvalue | per | Ratio |
| Group1 | DTS_108T/J806_ | - | - | | | | |
|  | DTS_123/TJ806 | - | - | | | | |
|  | DTS_127/TJ806 | - | - | | | | |
| Group2 | TJ806_AR02 | GO:0016829 | lyase activity | 120 | 1.37E-05 | 4.756 | 0.19 |
|  |  | GO:0044255 | cellular lipid metabolic process | 196 | 0.003196218 | 7.964 | 0.155 |
|  |  | GO:0006629 | lipid metabolic process | 252 | 0.003196218 | 10.24 | 0.148 |
|  |  | GO:0005506 | iron ion binding | 84 | 0.00337333 | 3.329 | 0.185 |
|  |  | GO:0003824 | catalytic activity | 1571 | 0.00978779 | 62.267 | 0.121 |
|  |  | GO:0071944 | cell periphery | 560 | 0.010006249 | 21.481 | 0.131 |
|  |  | GO:0048046 | apoplast | 75 | 0.010006249 | 2.877 | 0.179 |
|  |  | GO:0005886 | plasma membrane | 468 | 0.010006249 | 17.952 | 0.133 |
|  |  | GO:0005576 | extracellular region | 246 | 0.010006249 | 9.436 | 0.142 |
|  |  | GO:0016835 | carbon-oxygen lyase activity | 51 | 0.02450198 | 2.021 | 0.197 |
|  | TJ806_AR03 | GO:0016829 | lyase activity | 117 | 0.00042887 | 4.503 | 0.185 |
|  |  | GO:0003824 | catalytic activity | 1627 | 0.00174045 | 62.625 | 0.125 |
|  |  | GO:0004553 | hydrolase activity, hydrolyzing O-glycosyl compounds | 97 | 0.004344749 | 3.734 | 0.181 |
|  |  | GO:0016798 | hydrolase activity, acting on glycosyl bonds | 106 | 0.010235124 | 4.08 | 0.172 |
|  |  | GO:0016830 | carbon-carbon lyase activity | 50 | 0.028137548 | 1.925 | 0.201 |
|  |  | GO:0016758 | transferase activity, transferring hexosyl groups | 113 | 0.028137548 | 4.349 | 0.164 |
|  |  | GO:0033840 | NDP-glucose-starch glucosyltransferase activity | 5 | 0.028137548 | 0.192 | 0.833 |
|  |  | GO:0048046 | apoplast | 76 | 0.032338286 | 2.821 | 0.181 |
|  |  | GO:0031224 | intrinsic component of membrane | 768 | 0.032338286 | 28.508 | 0.13 |
|  |  | GO:0005576 | extracellular region | 250 | 0.032338286 | 9.28 | 0.145 |
|  | AR02_AR03 | GO:0060151 | peroxisome localization | 5 | 0.028731078 | 0.646 | 0.5 |
|  |  | GO:0043687 | post-translational protein modification | 5 | 0.028731078 | 0.646 | 0.455 |
|  |  | GO:0000146 | microfilament motor activity | 4 | 0.046064577 | 0.524 | 0.571 |
| Group3 | DTS108/DTS123 | GO:0071554 | cell wall organization or biogenesis | 15 | 0.00380855 | 18.75 | 0.014 |
|  |  | GO:0016798 | hydrolase activity, acting on glycosyl bonds | 11 | 0.004386542 | 13.253 | 0.018 |
|  |  | GO:0004553 | hydrolase activity, hydrolyzing O-glycosyl compounds | 10 | 0.004386542 | 12.048 | 0.019 |
|  |  | GO:0071555 | cell wall organization | 12 | 0.007533334 | 15 | 0.016 |
|  |  | GO:0045229 | external encapsulating structure organization | 12 | 0.008970085 | 15 | 0.015 |
|  | DTS108/DTS127 | GO:0005515 | protein binding | 3 | 0.023763864 | 75 | 0.001 |
|  |  | GO:0071944 | cell periphery | 4 | 0.033448502 | 66.667 | 0.001 |
|  | DTS123/DTS127 | GO:0005871 | kinesin complex | 3 | 0.026606043 | 6.522 | 0.042 |
|  |  | GO:0005856 | cytoskeleton | 8 | 0.026606043 | 17.391 | 0.009 |
| Group4 | DTS_108/AR02 | GO:0016829 | lyase activity | 122 | 0.000122271 | 4.552 | 0.193 |
|  |  | GO:0070192 | chromosome organization involved in meiotic cell cycle | 19 | 0.000458901 | 0.723 | 0.442 |
|  |  | GO:0007129 | synapsis | 15 | 0.000458901 | 0.571 | 0.517 |
|  |  | GO:0045143 | homologous chromosome segregation | 16 | 0.000641016 | 0.609 | 0.471 |
|  |  | GO:0044255 | cellular lipid metabolic process | 208 | 0.000724754 | 7.918 | 0.165 |
|  |  | GO:0006629 | lipid metabolic process | 265 | 0.002264519 | 10.088 | 0.155 |
|  |  | GO:0010333 | terpene synthase activity | 17 | 0.002452476 | 0.634 | 0.405 |
|  |  | GO:0005506 | iron ion binding | 87 | 0.003226354 | 3.246 | 0.192 |
|  |  | GO:0016798 | hydrolase activity, acting on glycosyl bonds | 111 | 0.003226354 | 4.142 | 0.18 |
|  |  | GO:0003824 | catalytic activity | 1668 | 0.003587354 | 62.239 | 0.128 |
|  | DTS_123/AR02 | GO:0016829 | lyase activity | 118 | 0.000220655 | 4.549 | 0.187 |
|  |  | GO:0015925 | galactosidase activity | 21 | 0.000635209 | 0.81 | 0.368 |
|  |  | GO:0016798 | hydrolase activity, acting on glycosyl bonds | 111 | 0.00095178 | 4.279 | 0.18 |
|  |  | GO:0004553 | hydrolase activity, hydrolyzing O-glycosyl compounds | 98 | 0.001754533 | 3.778 | 0.182 |
|  |  | GO:0048046 | apoplast | 80 | 0.003262605 | 2.99 | 0.191 |
|  |  | GO:0005506 | iron ion binding | 83 | 0.006941922 | 3.2 | 0.183 |
|  |  | GO:0048037 | cofactor binding | 130 | 0.008035887 | 5.012 | 0.165 |
|  |  | GO:0010333 | terpene synthase activity | 15 | 0.010467836 | 0.578 | 0.357 |
|  |  | GO:0016705 | oxidoreductase activity, acting on paired donors, with incorporation or reduction of molecular oxygen | 89 | 0.0177942 | 3.431 | 0.173 |
|  |  | GO:0004312 | fatty acid synthase activity | 17 | 0.01907736 | 0.655 | 0.309 |
|  | DTS_127/AR02 | GO:0016829 | lyase activity | 125 | 3.15E-06 | 4.808 | 0.198 |
|  |  | GO:0010333 | terpene synthase activity | 18 | 0.00028474 | 0.692 | 0.429 |
|  |  | GO:0016838 | carbon-oxygen lyase activity, acting on phosphates | 19 | 0.001665062 | 0.731 | 0.365 |
|  |  | GO:0071944 | cell periphery | 587 | 0.002302564 | 21.733 | 0.137 |
|  |  | GO:0048046 | apoplast | 80 | 0.002302564 | 2.962 | 0.191 |
|  |  | GO:0016841 | ammonia-lyase activity | 11 | 0.004633033 | 0.423 | 0.5 |
|  |  | GO:0016798 | hydrolase activity, acting on glycosyl bonds | 107 | 0.005075342 | 4.115 | 0.174 |
|  |  | GO:0006629 | lipid metabolic process | 259 | 0.005438379 | 10.217 | 0.152 |
|  |  | GO:0044255 | cellular lipid metabolic process | 198 | 0.006966131 | 7.811 | 0.157 |
|  |  | GO:0005886 | plasma membrane | 483 | 0.009460398 | 17.882 | 0.137 |
|  | DTS_108/AR03 | GO:0016829 | lyase activity | 125 | 7.94E-05 | 4.557 | 0.198 |
|  |  | GO:0004553 | hydrolase activity, hydrolyzing O-glycosyl compounds | 106 | 0.000457912 | 3.864 | 0.197 |
|  |  | GO:0046658 | anchored component of plasma membrane | 52 | 0.001046488 | 1.837 | 0.239 |
|  |  | GO:0003824 | catalytic activity | 1715 | 0.00105793 | 62.523 | 0.132 |
|  |  | GO:0016798 | hydrolase activity, acting on glycosyl bonds | 115 | 0.001249064 | 4.192 | 0.187 |
|  |  | GO:0006952 | defense response | 262 | 0.003497306 | 9.791 | 0.16 |
|  |  | GO:0044255 | cellular lipid metabolic process | 209 | 0.003497306 | 7.81 | 0.165 |
|  |  | GO:0006629 | lipid metabolic process | 269 | 0.003997945 | 10.052 | 0.158 |
|  |  | GO:0008610 | lipid biosynthetic process | 162 | 0.003997945 | 6.054 | 0.171 |
|  |  | GO:0070192 | chromosome organization involved in meiotic cell cycle | 17 | 0.004259759 | 0.635 | 0.395 |
|  | DTS_123/AR03 | GO:0016829 | lyase activity | 113 | 0.000524351 | 4.531 | 0.179 |
|  |  | GO:0006952 | defense response | 248 | 0.00078796 | 10.127 | 0.151 |
|  |  | GO:0004553 | hydrolase activity, hydrolyzing O-glycosyl compounds | 93 | 0.01081474 | 3.729 | 0.173 |
|  |  | GO:0016798 | hydrolase activity, acting on glycosyl bonds | 102 | 0.017909254 | 4.09 | 0.166 |
|  |  | GO:0016830 | carbon-carbon lyase activity | 49 | 0.02556223 | 1.965 | 0.197 |
|  |  | GO:0016491 | oxidoreductase activity | 311 | 0.026456179 | 12.47 | 0.137 |
|  |  | GO:0033840 | NDP-glucose-starch glucosyltransferase activity | 5 | 0.026456179 | 0.2 | 0.833 |
|  |  | GO:0015925 | galactosidase activity | 17 | 0.026691897 | 0.682 | 0.298 |
|  |  | GO:0004557 | alpha-galactosidase activity | 9 | 0.026786174 | 0.361 | 0.45 |
|  |  | GO:0052692 | raffinose alpha-galactosidase activity | 9 | 0.026786174 | 0.361 | 0.45 |
|  |  | GO:0003824 | catalytic activity | 1541 | 0.027308912 | 61.788 | 0.119 |
|  |  | GO:0043546 | molybdopterin cofactor binding | 8 | 0.033963387 | 0.321 | 0.471 |
|  |  | GO:0015166 | polyol transmembrane transporter activity | 11 | 0.033963387 | 0.441 | 0.367 |
|  |  | GO:0015168 | glycerol transmembrane transporter activity | 10 | 0.038151979 | 0.401 | 0.385 |
|  | DTS_127/AR03 | GO:0016829 | lyase activity | 125 | 9.36E-05 | 4.544 | 0.198 |
|  |  | GO:0003824 | catalytic activity | 1732 | 0.000119787 | 62.959 | 0.133 |
|  |  | GO:0071944 | cell periphery | 617 | 0.00069426 | 21.718 | 0.144 |
|  |  | GO:0048046 | apoplast | 85 | 0.00069426 | 2.992 | 0.203 |
|  |  | GO:0005576 | extracellular region | 273 | 0.001166659 | 9.609 | 0.158 |
|  |  | GO:0016491 | oxidoreductase activity | 351 | 0.002088676 | 12.759 | 0.154 |
|  |  | GO:0006952 | defense response | 264 | 0.002747176 | 9.869 | 0.161 |
|  |  | GO:0046658 | anchored component of plasma membrane | 49 | 0.003218213 | 1.725 | 0.225 |
|  |  | GO:0031224 | intrinsic component of membrane | 813 | 0.004835962 | 28.617 | 0.138 |
|  |  | GO:0016830 | carbon-carbon lyase activity | 55 | 0.005157744 | 1.999 | 0.221 |

| Table S11. Pairwise compositional comparison of DAMs in different maize lines. | | | | | | | |
| --- | --- | --- | --- | --- | --- | --- | --- |
| Group | Comparisons | Total | DAMs raito of detected metabolite(%) | Up-regulated in former | Ratio | Down-regulated in former | Ratio |
| Group1 | DTS_108/TJ806 | 11 | 0.56% | 6 | 54.55% | 5 | 45.45% |
|  | DTS_123/TJ806 | 40 | 2.05% | 21 | 52.50% | 19 | 47.50% |
|  | DTS_127/TJ806 | 19 | 0.97% | 10 | 52.63% | 9 | 47.37% |
| Group2 | AR02/AR03 | 16 | 0.82% | 11 | 68.75% | 5 | 31.25% |
|  | AR02/TJ806 | 65 | 3.33% | 20 | 30.77% | 45 | 69.23% |
|  | AR03/TJ806 | 129 | 6.60% | 68 | 52.71% | 61 | 47.29% |
| Group3 | DTS_108/DTS_123 | 14 | 0.72% | 3 | 21.43% | 11 | 78.57% |
|  | DTS_108/DTS_127 | 6 | 0.31% | 0 | 0.00% | 6 | 100.00% |
|  | DTS_123/DTS_127 | 11 | 0.56% | 8 | 72.73% | 3 | 27.27% |
| Group4 | DTS_108/AR02 | 32 | 1.64% | 7 | 21.88% | 25 | 78.13% |
|  | DTS_123/AR02 | 28 | 1.43% | 12 | 42.86% | 16 | 57.14% |
|  | DTS_127/AR02 | 15 | 0.77% | 10 | 66.67% | 5 | 33.33% |
|  | DTS_108/AR03 | 65 | 3.33% | 11 | 16.92% | 54 | 83.08% |
|  | DTS_123/AR03 | 76 | 3.89% | 22 | 28.95% | 54 | 71.05% |
|  | DTS_127/AR03 | 41 | 2.10% | 12 | 29.27% | 29 | 70.73% |

| Table S12. KEGG pathway enrichment analysis of significantly shared or specific DAMs to different comparisons. | | | | | | |
| --- | --- | --- | --- | --- | --- | --- |
| Comparisions | Group | KEGG | Total | Expected | Hits | Raw p |
| Group1/Group2 | Specific DAMs of Group2 | Vitamin B6 metabolism | 9 | 0.101 | 2 | 0.004 |
|  | Specific DAMs of Group1 | - | | | | |
|  | Shared DAMs of Group1 and Group2 | - | | | | |
| DTS_108/TJ806 vs Group2 | Specific DAMs of DTS_108/TJ806 | - | | | | |
|  | Specific DAMs of Group2 | - | | | | |
|  | Shared DAMs of DTS_108/TJ806 and Group2 | - | | | | |
| DTS_123/TJ806 vs Group2 | Specific DAMs of DTS_123/TJ806 | - | | | | |
|  | Specific DAMs of Group2 | - | | | | |
|  | Shared DAMs of DTS_108/TJ806 and Group2 | - | | | | |
| DTS_127/TJ806 vs Group2 | Specific DAMs of DTS_127/TJ806 | - | | | | |
|  | Specific DAMs of Group2 | - | | | | |
|  | Shared DAMs of DTS_108/TJ806 and Group2 | - | | | | |
